# Current audiological diagnostics

**DOI:** 10.3205/cto000148

**Published:** 2017-12-18

**Authors:** Sebastian Hoth, Izet Baljić

**Affiliations:** 1 Functional Area of Audiology, Department of Otolaryngology, University of Heidelberg, Germany; 2 Department of Otolaryngology, HELIOS Hospital of Erfurt, Germany

**Keywords:** hearing disorders, hearing tests, pure tone threshold, recruitment tests, speech audiometry, impedance audiometry, otoacoustic emissions, auditory evoked potentials

## Abstract

Today’s audiological functional diagnostics is based on a variety of hearing tests, whose large number takes account of the variety of malfunctions of a complex sensory organ system and the necessity to examine it in a differentiated manner and at any age of life. The objective is to identify nature and origin of the hearing loss and to quantify its extent as far as necessary to dispose of the information needed to initiate the adequate medical (conservative or operational) treatment or the provision with technical hearing aids or prostheses. Moreover, audiometry provides the basis for the assessment of impairment and handicap as well as for the calculation of the degree of disability. In the present overview, the current state of the method inventory available for practical use is described, starting from basic diagnostics over to complex special techniques. The presentation is systematically grouped in subjective procedures, based on psychoacoustic exploration, and objective methods, based on physical measurements: preliminary hearing tests, pure tone threshold, suprathreshold processing of sound intensity, directional hearing, speech understanding in quiet and in noise, dichotic hearing, tympanogram, acoustic reflex, otoacoustic emissions and auditory evoked potentials. Apart from a few still existing gaps, this method inventory covers the whole spectrum of all clinically relevant functional deficits of the auditory system.

## Abbreviations

aABR – Automated auditory brainstem responses

ABR – Auditory brainstem responses

AEP – Auditory evoked potentials

ABLB – Alternate binaural loudness balance

ASSR – Auditory steady state responses

AS/AN – Auditory synaptopathy/neuropathy

BERA – Brainstem electric response audiometry

BILD – Binaural intelligibility level difference

CAP – Compound action potential

CCITT – Comité Consultatif International Téléphonique et Télégraphique

CERA – Cortical electric response audiometry

CI – Cochlear implant

C-HL – Conductive hearing loss

CM – Cochlear microphonics

dB – Decibel

dB HL – dB hearing level

dB SL – dB sensation level

dB SNR – dB signal to noise ratio

dB SPL – dB sound pressure level

DPOAE – Distortion product otoacoustic emissions

EAEP – Early auditory evoked potentials

eCAP – Electrically evoked compound action potential

ECochG – Electrocochleography

EOAE – Evoked OAE

ERA – Electric response audiometry

ERP – Event-related potentials

FF – Free field

Hz – Hertz

IHC – Inner hair cells

MdE – Reduction of earning capacity (Minderung der Erwerbsfähigkeit)

MMN – Mismatch negativity

OAE – Otoacoustic emissions

OHC – Outer hair cells

SAEP – Slow auditory evoked potentials

SISI – Short increment sensitivity index

SN-HL – Sensorineural hearing loss

SNR – Signal to noise ratio

SP – Summation potential

SRT – Speech reception threshold

TEN – Threshold equivalent noise

TEN(HL) – Threshold-equalizing noise hearing level

TEOAE – Transient evoked otoacoustic emissions

VRA – Visual reinforcement audiometry

## 1 Introduction

The reasons for allotting a comparably high interest to hearing are numerous and manifold. In its function as receiver of signals, the ear is equipped with outstanding properties that surprise technically interested people, in its function as biological system it fascinates scientists due to its different components and mechanisms, in its role as important element of differentiated communication it is one of the essential characteristics of humans, and as the sense, which is most directly related to the emotional level, it contributes significantly to the well-being of people. Consequently, functional disorders immediately lead to severe disadvantages in many domains of human life. Due to the extraordinary importance of hearing, telecommunication and entertainment electronics using audio reproduction meanwhile cover one of the industry branches with the highest turnover worldwide.

In parallel to the high importance of this precise and sensitive signal detector, hearing is also very susceptible to damage by different noxious substances. If we did not know better, the existence of a sensory organ like the human ear would be considered as impossible: the structure of its sensory cells on the one hand are able to react on pressure changes with deflections of atomic dimensions corresponding to an altitude difference of 1.5 µm in the earth’s atmosphere; on the other hand it is sufficiently robust regarding vibrations and differences in height of several 100 or 1000 m. To compensate these requirements, evolution chose embedding this sensory organ in the most solid bone of the skeleton as well as a highly sophisticated mechanism for pressure transmission and a refined supply system. The subsequent level of signal processing involves very complicated and thus efficient procedures in the structures of the ascending hearing pathways and the cerebral cortex. Monitoring, maintenance, and repairing – if necessary – of this complex signal-processing system, is the task of ENT specialists who are the ones disposing of the instruments of functional auditory diagnostics.

Audiometry allows identifying and quantifying hearing disorders. Differentiated and quantitative assessment of the hearing capability aims at providing all necessary data which are needed for the choice of the adequate conservative, surgical, technical, or rehabilitative therapy. It seems to be obvious that the clear identification of the nature and severity of the hearing disorder cannot be done with one single functional test. Functional disorders of the hearing system occur in relation to all dimensions of the sound signal – frequency and intensity, time and space. Accordingly, the deficits are not or only partly mirrored in the “hearing test”, i.e. the lowest level of perception of pure tones (hearing threshold). The strength of current audiometry consists of the multitude of methods and their interrelations.

Recently, the basic knowledge of audiology could be enormously enlarged, the technology and application spectrum of technical hearing aids made extraordinary advances, and the possibilities of ear surgery grew impressively due to experimental evaluations and theoretical modelling of the middle ear apparatus. Dealing with publicly available knowledge and treatment modalities is no longer a facultative option of the users but an obligation. This fact justifies the high requirements to audiological competence in ENT practice. This present contribution aims to describe the current status of audiological functional diagnostics, however, despite its claim to be as exhaustive as possible, it will be limited to hearing tests applied in practice that are relevant for therapy planning.

## 2 Basics

It may be assumed that the readers of this article dispose of basic knowledge of physical as well as technical acoustics and of the anatomy and physiology of the hearing system. A short description of the most important terms, however, is essential to allow clarity of the explanations.

### 2.1 Acoustics

In the field of audiometry, the correct use of the parameters regarding sound signals is essential. The three acoustic dimensions are frequency, intensity, and time. There is only low need for explanation with regard to the frequency, which is measured in Hz (Hertz). For a periodic sound event this parameter defines the number of vibrations per second. Via the sound velocity, it is reciprocally connected with the wave length that has to be taken into account in audiometry only in the context of propagation of sound in limited spaces (examination room, sound tube, or auditory canal) – with the consequence of resonances or standing waves – as well as the effect of obstacles (“shadow” of the head in binaural hearing).

The intensity of an acoustic event describes the energy transported per time unit and area. It is measured in W/m² (Watt per square meter), a unit that is neither descriptive nor occurs in daily life. Between this unit and the amplitude or sound pressure measured in µPa (micropascal), there is a simple (quadratic) relation. It is due to the large dynamic range of the human ear (the range between the lowest perceptible and the highest tolerable intensity is divided into 12 orders of magnitude) and its functionality (high sensitivity for differences and changes at low intensities, low sensitivity at high intensities) that neither the parameters of intensity nor sound pressure are used but the calculated sound level measured in Decibel (dB) – named after Alexander Graham Bell. Perhaps this is the only physical unit that is exclusively used as tenth (“deci”).

In nearly all cases, the sound level requires a reference in addition to the dB measurement. “dB SPL” (sound pressure level) refers to 20 µPa (corresponding to the mean hearing threshold of the normal human hearing at 2 kHz) or “dB HL” (hearing level – not hearing loss) that corresponds to the physiologically normal threshold of the according frequency. Using dB without a reference is only allowed to describe differences (“hearing loss has increased by 10 dB”). A level of “0 dB” does not mean that there is no sound but it refers to the sound intensity given by the reference value, which is based on its definition as logarithmic measure so that an increase by 10 dB is accompanied by a 10-fold sound intensity or 100-fold sound amplitude (pressure or density), leading to approximately the double of the perceived loudness.

The time course of the sound signal is always important where (time-related) modulations occur, such as for example in cases of frequency-modulated wobble tones in free-field audiometry or amplitude-modulated test tones of the SISI test. In spoken language, the time modulations of amplitudes and frequency are the actual information carriers corresponding to a syllable frequency of around 4 Hz. This is taken into consideration by the use of modulated speech-simulating noise in speech audiometry [1].

In audiometric examinations, acoustic stimuli are provided via ear phones, bone vibrators, insert phones, or free-field loudspeakers. These technical devices are summarized as (electroacoustic) transducers. Their output signal can be measured in dB. Bone vibrators represent a special case because they do not cause a sound but a force level, which can be translated into a sensitivity level (dB HL) based on predefined technical requirements.

### 2.2 Anatomy and physiology of the hearing system

The sensory organ for perceiving sound waves is the organ of Corti in the inner ear. The signal reaches it via the air, the external ear, the external auditory meatus, the tympanic membrane, and the middle ear. From the inner ear, the stimulus triggered by the acoustic signal is processed, registered, interpreted, and classified via the neural structures of the hearing pathway (hearing nerve, brainstem, midbrain, thalamus, cortex, and association areas). During their course, the neural pathways of the right and left inner ear branch several times to the contralateral side so that information from both ears reach both brain hemispheres. This binaural processing of signals means that the right and the left ear are interrelated so that hearing must be considered as a singular sensory organ equipped with two peripheral “receivers”.

The middle ear is responsible for the low-loss transmission of vibrations into the inner ear. The apparatus consisting of tympanic membrane, ossicles, and tympanic cavity works as impedance transducer. Its effect is based on increasing the sound pressure as well as the deflection and thus velocity of the vibrations.

The vibrations transmitted by the stapes cause hydromechanic pressure variations in the perilymph of the cochlea including the cochlear partition and spread along the cochlear turns. Due to the location-depending physical properties of the liquid and the membrane, this transducing wave causes a local amplitude maximum in a place that depends on the frequency: coming from the cochlear base, the amplitude of the hydromechanic wave slowly increases until it suddenly collapses. In this way, high frequencies are processed at the base and low frequencies are processed at the apex.

The deflection of the cilia triggers active movements in the outer hair cells (OHC) of the organ of Corti located on the basilar membrane, and electric and chemical processes in the inner hair cells (IHC) leading to the discharge of neurotransmitters. The latter ones trigger the generation of action potentials by the corresponding fibers of the hearing nerve that spread afferently along the neural fibers. 

Functional or organic disorders may occur in nearly all structures of the hearing system [2], [3], [4]. According to the symptoms, it proves to be useful to categorize hearing disorders in the categories conductive, sensory, neural, and central impairments. Hereby, terms based on history such as sensorineural hearing loss or cochlear and retrocochlear hearing loss are also taken into consideration, but they are differentiated more systematically.

## 3 Subjective audiometry

If the preconditions are fulfilled, the assessment of the performance of the ear is best and most reliably examined by the evaluation of the patient’s subjective perceptions. Ideally, the perceptions are described in a qualified way, and, at the same time or exclusively, the patient and his reaction on the stimulus are observed attentively. In any case, subjective audiometry is not a measurement but a psycho-acoustic experiment that is based on the sensation triggered by the stimulus. This experiment has to be conducted under certain conditions that are defined in international standards of the series 8253 [5], [6], [7]. In contrast to objective hearing tests, it is not only the methodical procedure (“ask, not measure”) that is relevant but also the low specificity of the examination outcome regarding the anatomical structure responsible for the functional disorder.

### 3.1 Orienting hearing tests

The simplest method to get information about hearing loss is performing so-called orienting hearing tests (tuning fork and clinical hearing tests). In this way, the ear can be examined without any technical equipment, achieving a relatively low precision, however, a reasonable specificity.

#### 3.1.1 Tuning fork tests

Some reliable methods even from the time before introduction of the first valve audiometers for testing of the hearing capacities have survived until now. Those are for example some tuning fork tests. They do not provide quantitative measurements for the severity of hearing loss, but they give a certain orientation regarding its origin and nature. When placed on the skull, the vibrating tuning fork leads to direct stimulation of the inner ear via bone conduction. In the case of unilateral hearing loss, the perception is lateralized in a way that is specific for the pathology (Weber test): In cases of sensory or neural hearing loss, the patient hears the sound of the tuning fork in the healthy (better hearing) ear; in cases of conductive hearing loss, however, the sound is lateralized to the poorly hearing ear. This phenomenon can be explained by an amplification of the osteo-tympanic vibration component caused by the interrupted sound conduction pathway.

Another tuning fork test compares the sound perception via bone conduction with the one via air conduction (Rinne test): The tuning fork is first placed on the mastoid. As soon as it is no longer perceived, the examiner holds the fork in front of the entrance of the ear canal. A patient suffering from hearing loss in the inner ear (as well as a normally hearing person) can now hear the sound in contrast to a patient with conductive hearing loss. In contrast, if the sound of the tuning fork is perceived louder via bone

conduction (“behind the ear”) than in front of the ear (air conduction), a middle ear hearing loss of about 20 dB or more is present (this is also the situation when the tested ear is completely deaf, the contralateral ear is nearly normally hearing and neither the examiner nor the patient were aware of this). The results of Weber and Rinne tests cannot be clearly interpreted in cases of combined hearing loss; furthermore the Weber test is not relevant for bilateral hearing loss. See Table 1 (Tab. 1) and Table 2 (Tab. 2).

#### 3.1.2 Speaker distance examination

Since the sound pressure level decreases with growing distance from the sound source, understanding of whispering and daily language via the ear directed to the examiner can be assessed depending from the distance between the examiner and the patient. Words of four syllables (preferably numbers between 21 and 99) are whispered or spoken in normal daily pronunciation from various distances. When 3 or more of the whispered words are repeated correctly by the patient from a distance of 6 m or more, a middle or high-grade hearing disorder can most likely be excluded. The volume and the spectrum of the test signal are influenced by changing the distance to the tested ear (half of the distance means a higher sound level of around 5 dB) and by switching from whispering to normal pronunciation (the sound level of whispering is lower by 20 dB compared to daily language for the same distance and whispering contains significantly higher frequencies). A skillful examiner can roughly estimate the extent of hearing loss and derive information about the affected frequencies.

The largest distance for which at least three subsequent numbers are repeated correctly is noted as test result. In cases of relevant differences between the hearing thresholds of both sides in favor of the non-test ear, its auditory canal has to be closed or effectively masked by sound via ear phones (broad-band noise with 60 dB HL for whispering and 90 dB HL for normal speaking). Even if all mentioned rules are respected, the results obtained from speaker distance examination are not more than an orienting starting point for further diagnostics.

### 3.2 Pure tone audiometry

The attenuation component of a hearing impairment, i.e. the reduced sensitivity for sound signals of low intensity, is documented in a perception threshold for pure tones which is increased compared to normally hearing people. Its measurement is rather difficult because the most significant characteristic of the lowest perceptible level is the high uncertainty with regard to its perception. Thus the information given by the patient is generally limited regarding reliability and accuracy [8]. Another problem is that the threshold has to be determined for each ear but the patient usually has two hearing ears. Even if the test stimulus is delivered via earphones and not in the free sound field, the signal can be perceived by the contralateral ear. Only if the non-test ear is effectively masked, the threshold for the test ear can be determined exactly. The difference between the dB HL value of the measured hearing threshold and the normal values is called hearing loss.

Pure tone audiometry shows the thresholds between 125 Hz and 8 kHz in octaves and semi-octaves. For examination at higher frequencies, the frequency is increased in steps of sixths octaves up to 16 kHz (DIN EN 60645-1:2015-11). Measurement of the hearing threshold in the high frequencies, which is only possible with few standardized earphones (DIN EN 60645-1:2015-11), is only necessary and useful in the context of particular questions [9]. Those are for example the evaluation of the hearing abilities in the elderly [10], the early detection of ototoxic effects [11], [12] as well as the early detection of noise-induced hearing impairment [13], [14], [15]. A recent trial [16] also indicates that the high tone audiometry of most tinnitus patients with regular conventional pure tone threshold shows conspicuities. 

The complete pure tone audiometry contains graphs for air conduction and bone conduction. Air and bone vibrators are calibrated in a way that in case of intact physiological sound conduction both graphs are congruent (in contrast to Rinne test, which strictly speaking does not allow the conclusion that hearing via air conduction was “better”). The bone conduction threshold can only be measured reliably for frequencies above 250 Hz because at lower frequencies the bone conduction stimulus is not only heard but also felt before reaching the perception threshold (about 15 dB HL at 125 Hz). At frequencies between 250 and 1000 Hz, the threshold for tactile sensation is reached at hearing levels of around 40 dB HL at 250 Hz, 60 dB HL at 500 HZ, and 70 dB HL at 1000 Hz [6]. Depending on the construction of the bone vibrators, the definition of the bone conduction threshold may cause problems at high frequencies at or above 4 kHz because the bone vibrator may emit air sound that reaches and stimulates the ear via the sound transmission chain. In addition to these effects, in all frequencies, the stimulus intensity transmitted to the inner ear depends on the individual contact pressure and skin thickness and is thus less reproducible than for air conduction. The accuracy of bone conduction threshold is only about ±10 dB under the typical conditions of routine examinations.

During the last years, several trials have been published [17], [18], [19], [20], [21], [22], [23], [24], [25], [26] evaluating different mechanisms of sound transmission and perception via bone conduction. Among other results these studies revealed a significant correlation between the bone conduction threshold and the site of stimulation. The comparison of measurements showed that for determining the bone conduction threshold in clinical audiometry still the mastoid should be used as the most reliable site of stimulation [24], [27]. 

The conductive hearing loss that is apparent in the difference between air and bone conduction threshold (air-bone gap) never amounts to more than 50–60 dB because this corresponds to the amplification of the middle ear system and every sufficiently intensive air sound also causes mechanical vibration.

Stimulation of the ear via bone conduction is not side-specific because it causes vibration of the whole skull, nearly independent from the location of the transducer. If the bone vibrator is placed on the mastoid, the interaural attenuation amounts to max. 10 dB. Even when stimulating via air conduction, part of the vibration energy is transformed into bone vibration and thus stimulates not only the test ear but also the non-test contralateral ear. Regarding the closed supra-aural earphones mainly used in audiometry, the interaural attenuation amounts to about 50 dB, which can be improved to 80–90 dB with in-ear phones [28]. Perception in the non-test ear is avoided by masking this ear with narrowband noise via air conduction. Hereby, the effective level of masking in the non-test inner ear must exceed the level of the test sound. The necessary masking level can be calculated from the mentioned interaural attenuation and the sound conduction loss of the masked ear [1], [29], [30], [31]. In cases of unfavorable circumstances of air and bone conduction hearing loss in both ears, correct masking is not possible. A criterion that is valid without limitation for choosing the correct masking level, however, is that the threshold level increases isometrically with the masking level when the hearing threshold of the contralateral ear is measured instead of that of the test ear because of too weak masking. Only a sufficient contralateral masking stabilizes the threshold, i.e. it does not increase with higher masking levels. Attention must be paid that the masking noise is never over-dimensioned because this leads to over-masking and thus to a elevated threshold of the test ear.

According to its significance as a measure for reduced hearing perception, the audiometric hearing loss is a key criterion for the prescription of hearing aids. Based on §22 of the guidelines of the German Federal Joint Committee regarding the prescription of medical aids [32], the precondition for provision of unilateral hearing aids in the poorer ear is an audiometric hearing loss of at least 30 dB in at least one of the test frequencies between 500 and 4000 Hz. For binaural provision of hearing aids, the same conditions apply, however, referring to the better hearing ear (§ 21). 

In some areas, the hearing loss in percent plays an important role. It reduces the two-dimensional diversity of the audiometric hearing loss measured for many frequencies in dB to one single number given in percent which simplifies the situation for several purposes (even if the detailed information is lost). If the hearing loss at 1, 2, 3, and 4 kHz is known, the audiometry results included in the four frequency table [33], [34] provide a calculated percentage, for example a percentage of 95% in case of pancochlear hearing loss of 80 dB. Since the data from the four frequency table is generally much higher, especially in the context of noise-induced hearing loss, compared to speech audiometry [35], the more compatible three frequency table [36] is preferred nowadays (for the mentioned example, the percentage would then be 70%). It is based on the hearing thresholds measured at 1, 2, and 3 kHz that are included in the result with different weightings. Another table [37] in combination with the hearing loss percentage of both ears applies for the reduction of earning capacity (German: MdE, Minderung der Erwerbsfähigkeit). In cases of bilateral complete deafness, the reduction of earning capacity amounts to 80%. The procedure described here for assessing the amount of compensation applies for the statutory accident insurance based on the guidelines issued by the Federal Ministry of Labour and Social Affairs. Private accident insurances apply lower compensations of hearing impairment (60% for bilateral complete deafness) based on the dismemberment schedule according to the invalidity scale [38], [39]. See Table 3 (Tab. 3).

#### 3.2.1 Pure tone threshold in pediatric patients

The assessment of the hearing capacities in children is associated with several problems, like missing cooperation and the difficult evaluation of the reaction to acoustic stimuli, that do not occur in adults.

For testing of the hearing in newborns, only involuntary reflexes may be used that can only be triggered reliably with intensive stimulation beyond the hearing threshold. As of the 12^th^ week of life at the earliest, conscious reactions occur. Qualified and reliable data about the hearing threshold can only be expected in school children [30], [40].

Hearing tests in children require particular preconditions. Since younger children cannot be placed in an acoustic booth, a soundproof and acoustically isolated room is needed with an area of at least 12 m². The space should be free from excessive optical stimuli (possibly containing neither pictures nor toys) so that the children are not distracted from their actual task.

For free-field audiometry, the so-called Mainzer Kindertisch (children’s table) is necessary at which the child is placed (perhaps on its mother’s or father’s lap). On the table, loudspeakers are installed emitting wobble tones and noises. For distraction and rewarding, pictures are displayed (mostly on screens that are controlled via the audiometer) and other stimuli such as a moving train or lights at the loudspeakers. For this kind of audiometry, appropriate playing material is necessary (e.g. building blocks, stack towers etc.).

If the child sits on the mother’s or father’s lap during the test, careful attention must be paid to not influence the child by conscious (“You hear this noise, right?”) or unconscious contributions of the helper (turning to the source of the sound). The child should be awake and attentive (no examination late in the afternoon or after a long school day). Another precondition is a sensitive and patient examiner experienced in pediatric audiometry. The tests have to be performed without time pressure in a friendly and relaxed atmosphere. It is important to be open with the child and to appropriately explain the planned examinations.

Especially with restless or multiply handicapped children, it might be difficult to differentiate between incidental reactions and hearing reaction. Even experienced examiners seek advice of a colleague in order to discuss the child’s reactions. A good examiner also admits when he does not see clear hearing reactions and describes the reactions as “not evaluable”. Further examinations might then be necessary for assessing the hearing threshold. The child with hearing impairment does not benefit at all if its hearing is erroneously stated as too good by “favorable” assessment.

The subjective hearing tests are at least as important for the determination of the hearing threshold as the objective measurements. In the case of contradictory findings, one should rather rely on the results of the subjective tests.

In the context of audiometry, acoustic stimuli such as paper crackles, Orff instruments, rattles and whispering are offered and the child's reactions are observed. However, only an uncertain threshold definition is possible, since the stimuli are only imprecisely defined with regard to frequency range and sound level. Possible sources of error are vibrations, air draught or a simultaneous optical stimulus (e.g. rattles in front of the eye or the shadow of the examiner). Hearing impaired children can compensate for their handicap surprisingly well by using other sensory modalities.

In children up to the 12^th^ week, reflex reactions are observed for acoustic stimuli. The reactions are, for example, the Moro reflex (embracing reaction), the auropalpebral reflex (fast closing and opening of the eyes and contraction of the eyebrows), respiratory deceleration, pauses in nuckling or a fright reflex (e.g. crying). The acoustic stimuli can be offered via loudspeakers or via bone vibrators [41], [42]. The response threshold for these reflexes is far above the hearing threshold (in the first days of life up to 84 dB difference, decreasing to 16 dB at the end of the second year of life).

From the 6^th^ to 12^th^ week of life, not only unconditioned reflexes but specific reactions can be observed in the presence of childlike stimuli in the free-field. Signs of a hearing reaction are, for example, the change of breathing, sucking, mimics, or gesture. The reactions vary from child to child. Since both ears are always exposed in the free-field situation, a separate assessment of the ears is only possible to a very limited extent.

At the age of 6 months to 2 years, the reactions are clearer. Then the child can turn the head to the sound source or point to the loudspeaker [43]. Headphones are usually not tolerated at this age, but sometimes insert earphones are possible.

In conditioning audiometry, children from the age of 2 years onwards are offered a visual stimulus at the same time as a supra-threshold acoustic stimulus. In this way, the child should learn that both stimuli appear simultaneously (conditioning). In the subsequent hearing test, the visual stimulus is then activated later than the sound as a kind of reward. The level of the tone stimulus is then successively lowered in order to determine the hearing threshold.

For children from the age of 3 years, it is possible to train a game-like action that has to be performed when acoustic stimuli are perceived. For example, the child is asked to put a building brick into a box when it hears a sound.

From the age of 4 years, the examination can be performed separately for each ear by using headphones. If the child is able to make clear statements, it is also possible to test bone conduction by play audiometry in order to identify conductive hearing loss.

Determination of the hearing threshold with headphones and masking can be reliably performed from the age of 5 or 6 years. Then a child may be asked to press a manual switch when perceiving a sound stimulus, similar to audiometry in adults.

The hearing threshold of a regularly hearing child depends from its development and not necessarily from the age. In cases of retardation, normal hearing reactions may correspond to much younger children. All age spans given here should refer to the developmental age. According to our experience, the following values should apply to normal hearing [44]. See Table 4 (Tab. 4).

Probably the most important deficit of pure tone audiometry in neonates, infants and toddlers is that the threshold obtained in the test is higher or even significantly higher than the actual hearing threshold. This is all the more true the younger the child is (always with regard to its hearing developmental age). If we keep in mind that the perception of strong stimuli is not, or only slightly different, from that of normal hearing (recruitment), the difference between normal and impaired hearing is sometimes completely eliminated. If a newborn responds to an 80 dB stimulus, it can be normally hearing according to the above-mentioned table, but it is also possible that it is moderately to severely hearing impaired.

The discrepancy between the observed response threshold and the actual hearing threshold is reduced when the child's response is enhanced by attractive visual stimuli (illuminated and animated toys) [45], [46], [47]. In this way, reliable reactions become more likely because a small or very small child will probably show no interest for a non-moving colorful ball more than 2 or 3 times. Much more attractive are animals that can be seen behind a window and that wait for the child’s reaction being illuminated when activated, moving and playing funny instruments. If the reward stimulation is appropriately chosen, visual reinforcement audiometry (VRA) might obtain the result that the threshold of a normally hearing infant does no longer amount to 80 dB but to 20–30 dB. See Table 5 (Tab. 5).

### 3.3 Supra-threshold loudness sensation

Regarding the conversion of the sound intensity to an auditory perception, the hearing threshold is nothing more than the lower limit of the dynamic range. The upper limit of this area is the uncomfortable level (UCL) or loudness discomfort level (LDL) which should be part of the audiometric examination especially when provision of sound amplifying hearing aids is planned. Between the upper and the lower limit of the dynamic area, the loudness perception continuously increases according to the loudness growth function. As direct description of a possible dynamic compression, this function is suitable to differentiate a pathological recruitment. This is achieved with particular certainty in the binaural comparison of patients who are only hearing impaired in one ear. Less direct are the test paradigms of traditional “supra-threshold tests” that do not identify the recruitment itself but recruitment equivalents such as the ability to distinguish between the different levels of the hearing threshold in noise. Because of the increasing significance of objective audiometry and of imaging procedures, most supra-threshold tests became less important in the clinical practice over the last years. However, no other test could substitute the original test procedures for assessing the recruitment.

#### 3.3.1 Threshold of discomfort

The simplest option to audiologically assess individual changes of the perceptible dynamic area – which may occur due to recruitment, tinnitus, or hyperacusis – is to measure the discomfort level. In order to measure this supra-threshold parameter that in normally hearing people amounts to about 90 dB HL, different acoustic signals are offered separately via headphones – depending on the diagnostic question. In the clinical routine, mostly sinus tones of the frequencies from 0.5 kHz to 4 kHz are used. Their level – starting in the slightly supra-threshold range [48] – is increased gradually until the patient perceives the signal as uncomfortable. This tolerance limit which is considered as pathological for levels of less than about 80 dB HL [28] strongly depends on the instruction given to the patient [49] so that variations of the discomfort threshold of up to 20 dB [50] or more [51] are reported. Audiological standard manuals do not give clear instructions, for example Feldmann (1992) [52] or Goebel (2011) [53] recommend the following instruction based on Tyler and Conrad-Armes (1983) [54] and Vernon (1987) [55]. “Imagine that this was your radio or TV. Please indicate the loudness at which you would like to reduce the volume.” Goebel and Flötzinger (2008) [56], however, stated that the discomfort threshold is subject to a high intraindividual variability and thus it should only be applied as screening procedure in the context of diagnosing of hyperacusis [53]. Also for assessment of the recruitment [29] or in the context of hearing aids [30], [57], or for diagnosis of tinnitus [48], loudness scaling seems to provide more reliable results than the discomfort threshold measurement.

#### 3.3.2 Loudness scaling

Before starting a discussion about the term of loudness perception, this term has to be exactly defined. Psychoacoustics provide different concepts in order to solve this question that is not trivial at all [58], [59]. The difference is made between comparing and direct scaling and the effect of the spectral composition of the stimulus is described. The direct scaling of the loudness perception of narrow-band noise in dependence of the sound level was developed by Heller in 1985 [60], introduced in audiometry by Moser in 1987 [61] and is nowadays applied for clinical purposes in an internationally standardized [62] way [63], [64]. 

In this procedure, the patient scales his subjective loudness perception by categorizing the provided stimuli in verbal descriptions reaching from “not heard” to “extremely loud”. On a numeric scale, those values can be further categorized (0–50 units). Center frequency and level of the narrow-band pulses possibly cover the whole field of 250 Hz to 8 kHz; the stimuli are offered in a randomized way in the free-field or via headphones. For each tested frequency, the examiner receives a characteristic level-loudness curve of which the slope shows the recruitment. From the summary of those psychometric functions, isophones may be constructed and noted as individual hearing field in the audiogram.

The point of intersection of the single level-loudness functions with the x-axis is correlated to the hearing threshold (0 KU), the level described as “extremely loud” corresponds to the discomfort threshold (50 KU). If a pathological loudness growth function is found, the characteristic line is very steep, i.e. the horizontal distance between the individual curve and the normal value decreases with increasing level [64]. This horizontal distance corresponds to the level- and frequency-related amplification requirement that may be used as basic value for the amplification and compression of a hearing aid [57], [65], [66], [67], [68]. The loudness scaling can also be used as relatively sensitive procedure in the context of audiological topodiagnostics [66], [69], [70], [71], [72], [73]. See Table 6 (Tab. 6).

#### 3.3.3 Fowler test

The recruitment can be diagnosed in a simple and reliable way when a patient suffers only from unilateral hearing impairment, i.e. the other ear is normally or nearly normally hearing. The Fowler test or ABLB test (alternate binaural loudness balance) provides an interaural comparison of the loudness [74]. The patient is subject to sounds of the same frequency and variable intensity alternating for both ears. For every pair, the level of the better hearing ear is readjusted until the patient perceives the sounds in both ears with the same loudness. Those levels are registered in the audiogram and connected with lines. If the lines are parallel for all levels, no loudness equalization is present; however for confluent lines, the recruitment test is positive: the patient perceives high sound intensities in the hearing impaired and the normally hearing ear as equally loud. Those findings correspond to the pathognomonic statement of an endocochlear hearing disorder associated with a limited dynamic range. According to Kießling et al. [71], with regard to the determination of recruitment, Fowler’s test is highly sensitive compared to the other supra-threshold tests including loudness scaling. See Table 7 (Tab. 7).

#### 3.3.4 SISI test

For the diagnosis of recruitment, also the SISI test is used (short increment sensitivity index) [75]. The target parameter is the percentage of correctly recognized 1 dB increments with which a supra-threshold continuous tone is modulated. The correlation between this parameter and the pathological loudness growth function is obvious: because of the steeper input/output function, a given level difference in the ear with hair cell damage corresponds to a larger increase of the subjectively perceived loudness compared to the normally hearing ear and may thus be better recognized.

To perform the SISI test, a test frequency is chosen at which the hearing loss amounts to at least 40 dB. For lower hearing impairment, the application of the test is not suitable because only a substantial functional loss of the outer hair cells leads to pathological loudness growth. The continuous tone is adjusted at 20 dB above the threshold. In regular intervals of 5 seconds, the level is automatically increased for 0.2 seconds. For conditioning of the patient, 5 dB increments are offered, then the actual test starts with 1 dB increments. If the patient perceives at least 70% of those increments, it is considered as indication for recruitment and thus a cochlea-related hearing loss. Negative test results (less than 15%) speak against inner ear damage and indicate a neural origin of the hearing loss (e.g. depletion of hearing nerve fibers). Recognition rates between 20 and 65% should be interpreted carefully since they could be due to insufficient cooperation of the patient or inappropriate test parameters (frequency and intensity of the stimulus) [29], [30], [31]. Because of the unspecified variety of results, the diagnostic relevance of the SISI test is classified as being rather low and it should be performed in combination with further tests (Fowler and Langenbeck tests, loudness scaling) in cases of topodiagnostic questions [70], [76], [77]. See Table 8 (Tab. 8).

#### 3.3.5 Lüscher test

The ability to perceive intensity changes can also be determined by the Lüscher test which is different from SISI test regarding its performance but the statements and results are similar [78]. In this test, it is not counted how often the patient correctly identifies a small and defined level difference but the just noticeable level difference is determined by reducing the increments of the continuous tone from 4 dB adjusted to 20 dB SL (sensation level, i.e. dB related to the individual hearing threshold) down to 0.2 dB until the patient does no longer perceive loudness variations [29], [30], [31]. For classification of the findings, the level difference threshold of 1 dB serves as basis. While values of ≤1 dB are considered as indicator for cochlear pathogenesis of the hearing loss, the origin for values of >1 dB is expected to be a retrocochlear process. Kießling et al., however, showed that the selection based on this threshold value does not allow for a significant differentiation [71]. Even if the Lüscher test is more sensitive than the SISI test, the practical relevance of both tests is similar [71].

#### 3.3.6 Langenbeck test

Noise audiometry according to Langenbeck is another method by which a recruitment equivalent can be identified. In this test, the hearing threshold of a sinus tone is determined for several frequencies masked by narrowband noise [79]. The perception of a signals in a noisy environment is a task which is very similar to the perception of small and short-term loudness growth. Therefore, by measuring the masked threshold, a pronounced sensitivity for level differences can be detected. The principle of the Langenbeck test is to measure this threshold at several frequencies with differently pronounced hearing loss. As a result, the level distinction thresholds of damaged and non-damaged regions of the inner ear can be compared with one another. The test is suitable for ears with a sensorineural high-frequency attenuation, as far as the hearing threshold is approximately normal in the low-frequency range [29], [30], [31].

The level of the masking broadband or narrowband noise is adjusted at a defined value between 45 and 75 dB according to the audiogram. Lower levels are not useful from a physiological point of view (operational range of the outer hair cells), higher levels may lead to hearing fatigue and thus to invalid results. Third octave band noise is first mixed with an inaudible sinus tone of low frequency. The test tone level is gradually increased and the perception threshold indicated by the patient is registered. After increasing the sound frequency (along with the center frequency of the noise), the next masked threshold is determined. When the frequencies approach to the area affected by the hearing loss, it does not have an effect on the location of the masked threshold in cases of pathological loudness growth. This convergence of the masked threshold into the threshold in quiet is the characteristic parameter of a sensory hearing loss. In cases of neural hearing impairment, however, the masked threshold deviates from the threshold in quiet, i.e. the masked threshold is increased in the frequency range affected by the hearing loss. This phenomenon can be expected and is plausible when the retrocochlear disorder is due to a significant depletion of the hearing nerve fibers so that the capacity to transmit information is already exhausted with processing pure tones or noise close to the hearing threshold. 

The practical significance of the “noise audiometry according to Langenbeck” is generally considered as being low. Together with Lüscher test and Békésy audiometry [80], that will not be described here, noise audiometry according to Langenbeck is mentioned even in the “Königsteiner recommendations” only with the remark of “among others, the following procedures may be considered …”. See Table 9 (Tab. 9).

#### 3.3.7 TEN test

If the inner hair cells do not function properly in a clearly delineated area of the organ of Corti, the perception for the corresponding frequencies is selectively reduced within the so-called “dead region”. Steep notches in the audiogram indicate dead regions but they are not reliable since the characteristic of a dead region is that the perception of the test tone of the selectively “poor” frequency is falsified by cross hearing in the neighboring regions [81].

It is the objective of the TEN test according to Moore [82] to identify dead regions. This test is based on measurements of the masked threshold of a pure tone during masking with a special signal. The frequency of the TEN(HL) noise (threshold-equalizing noise hearing level) corresponds to the frequency dependence of the normal physiological hearing threshold [83]. The presentation level of this noise is adjusted to 10 dB above the audiometric threshold measured for the target frequency. If the masked threshold for a sound of this frequency lies at 10 dB or more above the level of the masker, the test tone is not detected at the appropriate location but in neighboring, better hearing areas of the cochlea. Thus a dead region at the location of the test frequency is considered as being proven. Those findings are the basis to think about frequency transposition when supplying hearing aids. If the masked threshold amounts to 10 dB or more above the level of the masker for all test tones, independent from the frequency, hearing impairment is rather due to neural or central origin (this is also the delineation against the Langenbeck test that interprets a large distance between the threshold in quiet and masked threshold as an indicator for neural damage). See Table 10 (Tab. 10).

#### 3.3.8 Tinnitus analysis

For patients affected by tinnitus, the application of classical subjective and objective procedures is most important – regardless if the tinnitus occurs as accompanying phenomenon of a disease or as main and isolated symptom. As a standard procedure, its frequency and loudness are evaluated in addition to audiometry. For frequency evaluation, the patient receives a comparable sound at 10 dB in the affected ear (in cases of unilateral deafness in the contralateral ear), the frequency is modified – starting with the frequency of highest hearing loss – until it is most similar to the tinnitus frequency [48], [52]. In the majority of the cases, the tinnitus is perceived as a tone of high frequency very close to the frequency of the largest audiometric hearing loss [84].

After determining the tinnitus frequency, the individual loudness of the tinnitus is assessed with pulsed sounds or with narrowband noise in small dB intervals. Independent from the subjective loudness perception, the tinnitus level is mostly not higher than 5–10 dB SL [48], [84].

The assessment of tinnitus masking, i.e. the combination of noise and level that makes the tinnitus “disappear” by masking [52] as well as residual inhibition (assessing if and how long the tinnitus remains suppressed after switching off the masking signal [52]) plays an important role for therapeutic application of noisers and tinnitus instruments. See Table 11 (Tab. 11).

### 3.4 Speech audiometry

According to Prof. Friedrich Bezold (1842–1908) [85], the language itself contains such a perfect composition of all possible sound complexes that one would have to invent it if it did not already exist. This sentence (author’s translation) emphasizes the necessity of speech audiometry even if spoken language does not seem to be an appropriate stimulus for differentiated examination of the hearing capacities. Due to its complexity, the use of speech stimuli would rather contribute to a concealment than to a clarification of the disease with respect to the kind of hearing disorders and its severity. However, if a hearing test aims at assessing the difficulties occurring in communication and the resulting handicap or at verifying the achieved rehabilitation of communication abilities after hearing aid provision, “hearing test with language” is essential [85].

The complexity of speech and its processing shows another difficulty. In contrast to pure tone audiometry, the result of the examination is influenced by other factors besides hearing ability, attention, and concentration. Those factors are memory span, working memory, mother tongue, vocabulary, and association abilities of the patient. Because of the multitude of the contributing factors, it is not possible to establish a universal speech discrimination test assessing all aspects of speech understanding. Thus, speech audiometry comprises many tests that vary according to their field of application and their significance. The common parameter of all speech tests is that they check the ability of the patient to perceive linguistic speech stimuli. If the speech perception depends on the sound level, a psychometric discrimination function (performance-intensity function) may be defined that reflects the test results and allows comparison of different test procedures [86].

The selection of the test material used in speech audiometry depends on the objective of the examination. Perception limited to only rudimentary performance can only be assessed if the test stimulus contains enough redundancy as for example in case of semantically reasonable sentences or numbers. The other extreme represent nearly redundancy-free monosyllabic test words. When the speech material is defined, the difficulty can still be influenced by offering response alternatives (closed test). In order to repeatedly perform the same test in the same patient without expecting falsification of the results due to learning effects, the material has to be classified in equivalent test lists. Some tests, however, require that one or two test lists are presented to the patient prior to the actual examination so that he might appropriately adjust to the examination (exclusion of training effects), which increases the accuracy of the test results [87], [88]. Independent of the aim of the examination, the test material should take into account a statistically representative distribution of the phoneme incidence in spoken language [89].

A group or list of test items stored on mostly digital recording media is presented via headphones or loudspeakers, optionally with simultaneous, predefined noise. The patient is invited to repeat the item (word or sentence) or to select the correct response out of a list of alternatives and the examiner counts the correct answers. The test result then consists of a percentage of correctly repeated test words depending on the absolute or relative speech level (related to the noise level). The correlation between stimulus level (or level difference) and the percentage of correct answers (speech recognition index) is called discrimination function. Its main properties may be described by two parameters with sufficient exactness: the level (or the relation between signal and noise) for which the probability of a correct answer amounts to 50% (speech recognition threshold, SRT), and the slope of the discrimination function at this point.

#### 3.4.1 Freiburg speech intelligibility test

In German speaking countries, the Freiburg speech intelligibility test introduced by Hahlbrock in 1953 [85], [90], [91] is still the mostly applied speech audiometric test and is considered as reliable standard for many applications [92], [93]. The test material consists of 10 groups of 10 two-digit numbers and 20 groups of 20 monosyllabic nouns whose composition is based on phonemic balance and perceptive equivalence. Below a certain speech level (10 dB SL for numbers and 15 dB SPL for monosyllables) normally hearing people do not understand any of the words. Increasing levels lead to a higher percentage of correctly understood words until it finally reaches 100% at about 30 dB SL (numbers) and 50 dB SPL (monosyllables). Normally hearing people reach a percentage of 50% recognition of numbers at a level of 18.5 dB SPL on the average, the slope at this point (speech recognition threshold, SRT) amounts to about 8% per dB [94]. Because of the high redundancy of numbers, the discrimination curve is very steep. Due to the same reason, hearing impairment only influences the location but not the shape of the discrimination curve. The impact of a hearing disorder can be completely described by the difference of the SRT assessed in a hearing impaired person and the normal value. This difference is called hearing loss for speech (sometimes also called parameter a_1_). Since the correct perception of low vocal frequencies is already sufficient for the recognition of numbers, this hearing loss correlates well with the pure tone threshold at low frequencies. For orientation, the hearing threshold at 500 Hz is considered. A pure high frequency hearing loss, however, has only a low impact on the discrimination curve for numbers.

Compared to numbers, the monosyllables curve is shifted in direction of higher levels and its course is flatter in case of hearing loss. Both aspects are due to missing redundancy and to the fact that for correct recognition of the test words not only loud vowels but also soft consonants are important. Hearing disorders do not only influence the location but also the shape of the discrimination curve. So it is not useful to characterize this curve only by its shift. Especially in the case of severe high frequency hearing loss, the curve is very flat because of the difficult recognition of high consonants. Often, no 100% understanding of monosyllables is achieved until the level of discomfort is reached, i.e. a discrimination loss is found. In rare cases, speech discrimination decreases at higher levels after having achieved a maximal value (“R curve” or roll-off). The discrimination loss and the speech intelligibility reached at a level of 65 dB SPL are the most important measurement parameters of the monosyllabic test.

The role of the Freiburg test in hearing aid provision is defined in the corresponding guideline [32]. Thus the prescription of hearing aids is based on the speech discrimination assessed by means of monosyllabic test words. The indication is given if the speech discrimination achieved at 65 dB SPL does not exceed 80%. If this value is not achieved in the better ear, binaural supply is indicated whereas non-achievement of the limit value in the poorer ear leads to unilateral supply. Provision and fitting aim at improving the monosyllables recognition at 65 dB SPL by at least 20% or, if not otherwise possible, the shift of the optimal monosyllabic understanding to exactly this sound level [95]. Also in the context of cochlear implantation (CI), the recognition of monosyllables is a fundamental parameter. Under the best conditions of conventional hearing aids, it should be at or below 40% [96]; the data of other authors range from 30 to 60%, while asymmetric hearing loss leads to separate indication rules. Furthermore, the Freiburg test has a high relevance in postoperative audiological control of CI patients [97], [98].

In addition to clinical diagnosis, the Freiburg test plays a major role in the context of reimbursement in cases of noise damage of the inner ear that is caused by work-related exposure to noise. The regulations are defined in the so-called Königstein recommendations (German: Königsteiner Empfehlungen) issued by the Association of the German Statutory Accident Insurance according to which the reduction in earning capacity is determined [99]. The calculations performed by means of standardized tables include the a_1_ value evaluated according to the already described speech recognition threshold and the “overall speech comprehension” calculated from the sum of the monosyllabic recognition at 60, 80, and 100 dB SPL [35]. The parameter of overall speech comprehension was modified by Feldmann because the assessment of mild hearing loss did not meet the requirements of the improved hearing aid technology. The recognition rates were then weighed with the factors 3, 2, and 1 so that the comprehension at moderate loudness contributes more to the result [39].

The Freiburg test has some disadvantages reported in the past as well as in some recently published articles [93]:

- Many of the test words are no longer used today [100].
- The test lists are phonemically neither representative nor balanced [89].
- The test lists are not equivalent with regard to the difficulty [101], [102], [103], [104].
- The accuracy of measurement and thus the sensitivity are only low [105].
- The test of speech recognition in noise is not supported [106], [107].
- The test procedure cannot be automated because the patient’s reply has to be evaluated by the examiner.
- The patient’s answer is only evaluated as correct or wrong, phoneme confusions are not evaluated.
- The missing announcement stimulus leads to an inappropriately high ratio of false answers (“attention test”).
- The missing announcement and the short duration of the stimulus may lead to problems in case of hearing aids with automatic gain control.

Because of these disadvantages, further speech intelligibility tests have been developed and introduced in practice. However, despite the quantity of those tests, none of them meets all the mentioned criteria nor the standard of 8253-5 [5]. So it seems to be obvious that there will always be several tests in speech audiometry that are applied as single measures or in combination based on the specific question – just as the Freiburg test which in reality consists of 2 tests [91], one test for testing the speech perception threshold and the other to test speech discrimination.

Possible alternatives to the Freiburg test might be the rhyme test according to von Wallenberg and Kollmeier (WaKo) [108] in combination with the Oldenburg sentence test (OSLA) [87], [109], [110], [111] or the Göttingen sentence test (GÖSA) [112], [113]. By means of the following specific parameters of those tests, some of the above-mentioned deficits are omitted:

- WaKo: introductory sentence announcing the stimulus item, closed response inventory, automatic procedure and evaluation of phoneme confusions, percentage of speech recognition and speech perception threshold with and without noise.
- OLSA and GÖSA: adaptive determination of SRT with and without noise.

The most important parameter of those newer tests is not the percentage of correct answers (depending on the stimulus level) but the speech recognition threshold. The SRT is defined as sound level for which 50% of the test items have been repeated correctly. Adaptive procedures are applied for the determination of SRT [114]: the presentation level is reduced if more than 50% of the words within a test sentence were correct and increased otherwise. The threshold determined on the basis of discrimination functions corresponds to the position of the inflection point. It is obvious that this method can only be applied for sentences which have the further advantage that the settling time of the hearing aid has less impact on the result because of the longer speech item. The SRT is considered as sensitive measure for improvement of speech intelligibility. In a less sensitive but more comfortable variation of the test, the aim is not 50% but 70% speech discrimination.

Compared to the traditional Freiburg test, the focus of modern speech audiometry is no longer directed to the speech intelligibility in % but to the parameter SRT measured in dB. Together with the test-specific slope of the discrimination function which is measured in “% per dB” (i.e. “what is the percentage of improvement of the speech intelligibility if the speech level is increased by 1 dB?”) different but comparable parameter describing the effect of a therapeutic intervention is obtained. Due to the well-known nature of logarithmic relationships it must be kept in mind that the measure of “dB” without any further specification is only suitable for differences (for example the threshold with and without hearing aid). Dealing with relative (related to the level of the noise) or absolute values without noise (SRT in a quiet environment), the speech level is measured in “dB SNR” (signal to noise ratio) or in “dB SPL” (sound pressure level). The transition from the traditional to the new tests does not cause any problem with regard to the measures. It is further facilitated by an overview where all tests are presented in a comparative way based on the example of indication and follow-up of hearing aid supply [94].

In the following, the three most common modern procedures of speech audiometry will be described. Apart from these tests also the HSM sentence test (according to Hochmair, Schulz, and Moser, 1997 [115]), which is mainly applied in the context of CI evaluation, the two-syllables rhyme test [116] as well as various logatome tests [117] are in use, however, only in special applications. See Table 12 (Tab. 12).

#### 3.4.2 Rhyme test according to von Wallenberg and Kollmeier (WaKo)

In the monosyllables rhyme test WaKo according to von Wallenberg and Kollmeier (1989) [108], the acoustic presentation of a monosyllabic test word is preceded by the invitation to “mark the word”. The patient then selects the supposedly presented word among 5 alternatives on an interactive display [108]. The alternatives belonging to each target word are chosen according to the principle of minimal pairs, the elements vary only with regard to the initial sound, the medial sound (vowel), or the final sound (for example: chin – thin – tin – bin). The lists of the original version comprised 72 words, the short but nonetheless sufficiently exact version according to Brand and Wagener (2005) of the test consists of 18 lists with 25 words each [118].

The presentation of the stimulus items of one list is performed at a constant level, the percentage of the speech intelligibility results from the number of correct answers after correction regarding the guess probability. In order to determine the maximal speech understanding or its complement (discrimination loss) and the necessary speech level of dB-opt, the measurement series are performed at different levels.

The WaKo test offers the possibility to evaluate phoneme confusions based on falsely understood or not correctly identified speech sounds. Thus mistakes of the patients with regard to acoustic and articulatory characteristics of speech can be identified and specified. Another characteristic of the rhyme test is the automatic setting which does not require the continuous presence and intervention of the examiner.

The discrimination function of the WaKo test reveals a slope of 6% per dB in its inflection point (in quiet and noisy environment). If needed, the disturbing noise is a stationary noise simulating speech according to CCITT [119]. It overlays the speech material with a signal to noise distance of –1 dB (initial consonant and medial vowel) or +4 dB (final consonant) and starts 2 s before starting the target word and ends about 1 s after its end [108]. In order to achieve results which are comparable with the Freiburg monosyllabic test, the test items should be presented at a level that is 20 dB lower [120]. The WaKo test in quiet environment is an alternative to the Freiburg test [120], [121]. Theoretically it is a possible option with regard to the prescription of hearing aids that might replace the Freiburg test [94]. See Table 13 (Tab. 13).

#### 3.4.3 Göttingen sentence test (GÖSA)

For a long time, the Marburg sentence test that had reached the status of a standard [122] was the only sentence test in the practice [123]. However, during the years it became obvious that it is inappropriate for the broad clinical use because of several severe disadvantages [113]. Due to this fact, Wesselkamp and colleagues developed the Göttingen sentence test (GÖSA) [113]. To establish this test, strict criteria were defined which among others lead to a high perceptive balance of the test lists and allowed the application for measurements in noise.

The GÖSA consists of 20 test lists with 10 sentences of daily life each, as for example “Is it time to go home?”. Those sentences have 3–7 words each. For measurements in noise, a speech simulating noise was generated by statistically overlaying words of the monosyllabic rhyme test [108], [124] recorded by the same speaker [112]. While the GÖSA in noise is characterized by a high slope of the discrimination function in the inflection point of about 20% per dB, the discrimination function in a quiet environment is clearly flatter with a slope of about 11% per dB [125]. In order to understand 50% of the test items in a quiet surrounding, normally hearing people need a level of about 20 dB SPL. In noise, they succeed at a speech-noise level difference of about –6 dB S/N. The measurement of the GÖSA is generally performed in an adaptive way [114] with a fixed noise level, and it leads to a reliable result with two test lists. A previous training is not necessarily required, however, repetition of a test list should be avoided.

Even if the test was developed carefully, especially with the aim that the test situation should resemble a real hearing environment as far as possible, some disadvantages could not be avoided. The repeated use of test lists in the same patient represents a real problem because of the high informational content of the test sentences [112]. Furthermore, the number of test lists is classified as being insufficient for some complex issues [94], [126], [127]. Rapid recording as well as unclear articulation make testing difficult in some patients with higher-grade hearing impairment [128] or in CI patients [129]. But in particular regarding CI, in many cases the advantage of daily routine test sentences is higher so that the GÖSA is applied intensively in the context of CI evaluation.

Recent investigations show that despite some disadvantages, similar speech intelligibility thresholds may be achieved with the GÖSA compared to the Freiburg test with numbers [120]. Even the proof of benefit of hearing aids in noise, of which the level should be 45 dB SPL, seems to be possible with this sentence test [94] so that it is acknowledged by the current directive for prescription of technical aids [32]. In addition, a recent evaluation could show that the GÖSA may be applied in the context of assessing the reduction of earning capacity [130]. This approach, however, could not be established up to now. See Table 14 (Tab. 14).

#### 3.4.4 Oldenburg sentence test (OLSA)

Since earlier approaches did not lead to the desired result, Wagener et al. [87], [109], [111] developed a test based on the experience with existing sentence tests that summarizes all positive parameters of those tests in order to meet the increasing requirements of German speech audiometry [111]. The basis for this test was the matrix test [131] developed by Hagerman (1982) in Swedish that was qualified because of its many positive properties [111].

The open Oldenburg sentence test (OLSA) resulting from this project contains – similar to the “Hagerman test” – syntactically correct sentences which are semantically not predictable. The sentences are composed of a name, a verb, a number, an adjective, and an object (e.g. “Doris draws nine wet chairs”) [94]. Each of these 5 words is taken from a list of 10 alternatives; the sentences are built randomly. This makes it improbable that a test sentence is recognized again and the number of possible test lists increases. By 30-fold overlaying of the randomly time-shifted speech material, an optimally masking stationary noise could be produced whose long-term spectrum corresponds to the test material [111]. The discrimination function of the OLSA shows a slope of 11% per dB in a quiet environment at the SRT and 17% per dB in noise so that especially the SRT in noise, which amounts to –7.1 dB S/N in normally hearing people, can be determined with high accuracy. In contrast, the SRT in quiet environment amounts to 20 dB SPL as also for the GÖSA. In order to exclude training effects as far as possible, each measurement should be preceded by a test list [87]. As the GÖSA, the measurement of the OLSA is generally performed in an adaptive way [114] with fixed noise level. Reliable results can be obtained with test lists of 30 sentences. In 2011, the OLSA was included in the directive for technical hearing aids so that the benefit of hearing aids in noise (adaptively at 45 dB SPL) can now be proven by this test beside the GÖSA [32].

Beside the high accuracy for determining the SRT in noise, which amounts to 0.5 dB for hearing impaired people [114], one of the most important advantages of the OLSA is the large number of test lists. In the context of complex questions that either require several measurements in one session or repeated measurements (such as for example comparing hearing aid or CI fittings) this test provides a nearly endless test material. In contrast, the test – especially because of the mentioned training effect – is rather time consuming. Fluctuations in concentration, occuring particularly in older patients, might lead to false results. Also CI users who achieve less than 75% at a level of 65 dB SPL in quiet do not show reliable results with the OLSA so that this test is not appropriate for this group [132]. Another limitation is found in high-grade hearing impaired patients where neither speech nor noise can be presented at an audible level with the usual technical devices [128]. In cases of only mild hearing impairment, the OLSA may be problematic since a signal to noise ratio around –6 dB is not part of the acoustic reality of the patients [95]. 

Even if the “modern” sentence tests may represent different hearing situations – as the presence of noise and different spatial arrangements of the sources of speech and noise – and thus quantify speech understanding, further methodic options are desirable for the sake of practical applicability [95]. See Table 15 (Tab. 15).

#### 3.4.5 Speech audiometry in children

In pre-school children, the speech audiometric tests used for adults can be applied only to a limited extent. Referring to the Freiburg test, this is particularly true for monosyllables, whereas the numbers may be part of the limited vocabulary of (hearing impaired) children [133]. Regarding the OLSA, the sentences composed of 5 words may overstrain the limited memory span of a child. Furthermore, the possibilities of speech audiometric examinations in children are limited by the rapidly decreasing concentration and the limited vocabulary. These facts are taken into account by adapting the examination methods and the selection of appropriate test material.

The pediatric Mainz speech test consists of 3 parts that are adapted to different ages and developmental stages [43]. All 3 parts consist of 5 groups of 10 test words. The test material is not phonemically balanced because the aim of the selection of the test words was mainly to build age-appropriate lists. In the first (and simplest) part, the 5 groups consist of only 10 different words (car, bear, bow-wow, train, clock, mama, egg, doll, ball, meow); so only the order of the words is different. The second part of the test consists of 25 different words that are partly the same as in the first part. Each test word appears in 2 of the 5 word groups. In the third part of the test, no repetitions are found, i.e. the 5 groups are built of 50 different words. The test result is the number or percentage of correctly recognized and repeated test items at a defined speech level.

A general problem in speech audiometry is that the patient has to repeat the test word so that the examiner knows if the word was correctly understood. This may lead to the phenomenon that possible dysfunctions of the speech-production system are included in the result of the hearing test [40]. This problem may be avoided, if the test person disposes of illustrations which he may show when recognizing a word. This principle is realized in the Göttingen pediatric speech intelligibility test [134], [135] which consists of 2 parts. “Göttingen I” is applied for children at the age of 3–4 years. “Göttingen II” is used for pre-school children at the age of 4–5 years. Both tests refer to the speech understanding with monosyllabic test words.

The Mainz and Göttingen children tests are not suitable for testing speech understanding in noise. Especially for this purpose, the Oldenburg pediatric sentence test (OLKISA) was developed from the Oldenburg sentence test (OLSA) by modifying the test material [110], [136]. It consists of pseudo-sentences with 3 words each (number, adjective, noun, e.g. “nine little cups” and is evaluated for primary school-age children. For the application in pre-school children, the Oldenburg Rhyme Test for Children has been conceived [137]. For this test, the child has to point to the perceived word (e.g. “sun”) on a picture where three alternatives (a nun, a gun, and a sun) are shown. Since the discrimination function of this test has only a low slope, it is suitable for determination of the speech intelligibility threshold in noise only in a modified version [138], [139].

In pediatric patients, speech audiometry is mainly applied for prescription and fitting of hearing aids and and later follow-up, because the supply of technical devices in hearing impaired children aims primarily to support speaking and development. Hyper- and hypostimulation may impede the success of the supply or lead to damage of the ear. By means of the functional gain or with objective methods, the success can be validated only to a limited extent, but since speech understanding depends on the speech level, they can be seen in the speech audiogram. The aim of hearing aids is to achieve a normalized speech discrimination curve or at least a normal level of 50% speech understanding. See Table 16 (Tab. 16).

### 3.5 Binaural hearing

Many tasks of the hearing system such as the detection of signals (especially speech) under acoustically difficult conditions (for example in noise, with competing signals, or in echoing environment) or the acoustic localization can only be fulfilled by hearing with both ears. Binaural hearing is more than the sum of its parts because the signals coming from both ears are not processed independently but connected to each other in the centers of auditory processing in the brain stem [140]. Without any doubt, binaural hearing is the natural hearing situation. First, it differs from monaural hearing by a lower perception threshold. The binaural hearing threshold measured in the free field is about 3 dB lower (because of the doubling of the processed sound energy) than the monaural threshold. In case of supra-threshold stimuli, the difference between monaural and binaural hearing is even higher. It amounts to about 10 dB, i.e. the addition of the second ear leads to doubling of the loudness. This effect is called binaural loudness summation.

Two other effects of binaural hearing are based on the processing of interaural intensity and time differences: the acoustic localization of sound sources (directional hearing) and the reconstruction of noisy signals (squelch effect). The acoustic signal originating from a source located outside the median plane reaches the auditory canal of one ear earlier and with a higher intensity than the other ear. The evaluation of weighted delayed coincidences in specialized neuron groups of the brain stem is based on these differences (interaural time difference, ITD; and interaural level difference, ILD) [141].

#### 3.5.1 Speech intelligibility in noise

Binaural hearing is crucial for perception and recognition of signals in noise. The binaural threshold for the recognition of a test item in noise is lower than the monaural threshold, the difference between both is called BILD (binaural intelligibility level difference). Depending on the spatial source location of signal and noise, the threshold difference amounts to about 10 dB in normally hearing persons.

In the context of hearing prosthesis, BILD is a measure for the effect of adding the second ear. The highest value of the parameter is expected in the configuration of S0N90 (speech from the 0° direction, noise from the 90° direction) when the 90° direction corresponds to the side with the better hearing ear. For measuring BILD, a speech intelligibility threshold is assessed in the poorer ear first without and then with hearing aid, and then the difference is calculated from both thresholds [57]. The directive for technical aids [32] requires a gain of 2 dB as proof of benefit in cases of unilateral hearing aid.

The improvement of signal recognition described by BILD is closely related to the ability of speech understanding in noise (cocktail party effect) which requires a functioning binaural system. The function of the binaural system may be affected seriously by even minor unilateral hearing disorders. The examination of the uni- or bilateral discrimination thresholds is thus suitable for quantitative description of the ability to follow conversations in noise or buzz. It has been seen that BILD is reduced in hearing impaired patients and also in normally hearing elderly test persons.

#### 3.5.2 Testing of directional hearing

The ability of spatial hearing and locating sound sources is a predominant consequence of binaural perception and processing of acoustic signals [142]. Furthermore, the outer ear plays a major role. According to its acoustic function, it is a filter whose transmission function depends on the direction and distance of the sound source in a frequency-specific manner. Reflection, shading, spreading, flection, interference, and resonance translate the spatial characteristics of the sound field to time properties whose central nervous representation evokes the impression of spatiality. The existence of two inputs, the crossing course of the hearing pathways, and the processing of the differences between both signals (binaural processor) is a precondition for another aspect of spatial hearing, the directional hearing. The location of the auricles at both sides of the head leads to the fact that in case of lateral sound the two input signals are different with regard to level, incidence, and tone quality. The different tone quality results from the fact that shading of the head is only effective for high frequencies. For the same reason, the interaural level differences are particularly large for high frequencies which are not deflected by the head (head shadow effect). They contribute to the lateralization of hearing as soon as they exceed 1 dB. Furthermore, the sound waves from sources that are outside the median plane reach the ears at different times (or different phases at the same time). The experimentally determined lower limit for perception of interaural time differences amounts to less than 30 µs in humans [142]. Time and level differences allow determination of the direction with an accuracy of 3–5°. This localization of the sound source, however, is not always unambiguous. All sources that are on the surface of an imagined cone radiating from the middle of the head, the axis of which corresponds to the line between both ears (cone of confusion), have nearly the same interaural differences. Different points on this cone vary only regarding the tone quality. Actually, in tests for directional hearing in the horizontal plane the most frequent confusions occur for directions which are equivalent regarding interaural time and level differences. A special case is the confusion between front and rear. Also the determination of the elevation, i.e. the localization of sound sources that are in the median plane (opening angle of the cone equals 180°) takes place without the support of interaural differences and thus it is rather uncertain.

Interaural time differences are only useful for the localization of sound sources when the duration of the signal is sufficiently short. This precondition is not fulfilled in permanent sounds or in case of echoes. In these cases, the hearing impression of both ears merges to one hearing event. Nonetheless, the sources of permanent stimuli or a short signal that is accompanied by an echo can be localized. This is due to the fact that the location of such a hearing event is mainly determined by the change of the sound pressure that reaches the ear first (“law of the first wavefront” or precedence effect). Of course, this important mechanism may fail in closed rooms where reflections and standing waves are present.

In order to test directional hearing, the test person is asked to identify the direction of a sound source which is located in the horizontal plane. Beyond this scheme, there is neither a clear standard nor a generally accepted convention for testing directional hearing. The described test setups vary with regard to the number of loudspeakers and their spatial arrangement, the nature of stimuli and noise as well as the assessment of the test persons’ answers. The results are graphically displayed either in polar diagrams – to show the directions – or as xy graph – with the advantage that the equality of sound and hearing direction are immediately seen. Among others, the numeric results are the confusion matrix, hit rate, and accuracy [143], [144], furthermore the effective rms error and the minimum audible angle.

The most important practical application of directional hearing tests is to prove the benefit of binaural hearing and especially of the second hearing aid. Because of the great variety of test setups, there are no standardized reference values. The directive for technical aids requires in an unspecific way that the provision of hearing aids for the poorer ear should lead to an improved directional hearing. It is justified to assume that the assessment of not only better but significantly better directional hearing can be performed more reliably in specialized institutions than in private ENT practices. See Table 17 (Tab. 17).

### 3.6 Auditory processing disorders

Conspicuities or deficits in the sound processing on higher levels of the hearing system, especially the localization of sound sources or speech understanding in noise going along with the absence of detectable organic defects and a normal non-verbal intelligence are classified as auditory processing disorders ([C = central] APD). APDs are observed as isolated parameter or in combination with other deficits (attention-deficit/hyperactivity syndrome (ADHS), dyslexia, speech development disorder, or lack of concentration). The suspicion of APD often appears in school children in the first school grades. In accordance with to the definition, the following basic auditory functions

- Localization and lateralization of acoustic events
- Auditory differentiation
- Temporal aspects of hearing
- Temporal integration: differentiation of temporal structures, recognition of time sequences, forward masking
- Ability of hearing and understanding of competing (speech) signals including dichotic hearing and understanding
- Ability of hearing and understanding of signals of limited quality

have to be differentiated from the following higher discrimination performances [145]:

- Phonologic consciousness including auditory analysis and synthesis
- Auditory attentiveness
- Auditory memory including long-term, short-term and working memory
- Speech intelligibility, speech understanding, and interpretation of the content.

Since it is currently not possible to identify disorders in the field of neuronal processing of the hearing pathways in a reliable and specific manner, it cannot be completely excluded that also primary cognitive deficits have to be taken into consideration for the definition of (C)APD [146]. According to the German consensus, the interpretation of auditory information belongs to auditory processing and perception [146], [147], however, British and American guidelines (BSA, ASHA) hold the point of view that superordinate cognitive processes are not imperatively responsible for incorrect auditory processing of acoustic signals [146]. Without any doubt, the disorder is far from being assessed in a standardized way. However, there is consensus that before applying particular diagnostic procedures adapted to CAPD and associated with auditory processing (frequency discrimination, directional hearing, speech understanding in noise, dichotic hearing) a peripheral hearing disorder must be excluded [146]. The instruments used for this purpose, especially pure tone audiometry, ABR, and OAE, are described in detail in the respective paragraphs of this review. The suggestions for APD-specific diagnostics are numerous and develop rapidly [148], [149], [150], [151], [152]. The authors do not feel competent enough to describe this complex field and refer to consensus papers and guidelines [153], [154], [155], [156] and other sources that have already been mentioned.

## 4 Objective audiometry

In contrast to subjective audiometry, which is based on psycho-acoustic methods, all procedures measuring physiological reactions that accompany the hearing process and are relevant for hearing itself are called objective audiometry. Compared to subjective procedures, the registered signals are much less subject to the influence, the attention, and the active cooperation of the test person. Among the reactions on acoustic stimuli, the physical properties of the tympanic membrane (impedance audiometry), the oto-acoustic emissions (OAE), and the electric processes occurring in hearing nerve, hearing pathways, and cortex (auditory evoked potentials) can be evaluated. Since all signals used for objective audiometry are overlaid by interferences, the accuracy is generally limited. For assessment of the hearing threshold, their application is mainly interesting in patients who are not able or not willing to cooperate. If the patient’s cooperation is sufficient, there is no more reliable, more accurate, and more rapid method than asking the patient about his subjective hearing. However, the objective procedures provide differential diagnostic statements that cannot be gained by subjective audiometry. With regard to identification of hearing disorders and the localization of their origin, objective audiometry cannot replace but rather complete subjective audiometry.

### 4.1 Impedance audiometry

The basis of impedance audiometry is the acoustic resistance which is excerted by the tympanic membrane against the incoming sound wave. The measurement of this impedance as a function of the atmospheric pressure and the frequency of the tone (tympanometry) allows a detailed description of the physical properties of tympanic membrane, middle ear, and ossicles. The measurement of the impedance during acoustic stimulation with tone pulses further allows the observation of physiological reactions (stapedius reflex or acoustic reflex).

At the interface between two media of different sound impedances, the sound waves are in general partially reflected. The measurement of the impedance is based on the close relation between reflection and impedance difference. Physically, the impedance of the middle ear depends on the mass (tympanic membrane, ossicles, sometimes secretions), on the friction (tympanum and inner ear), and the elasticity (tympanic membrane, middle ear tendons, air in the tympanum) of the anatomic structures involved in the vibration as well as the sound frequency. Most of the mentioned parameters are characteristic for the middle ear. At usual test frequencies, the most important and diagnostically most relevant contribution to impedance is the elasticity.

The relation between impedance and sound frequency is most relevant for the selection of the probe tone. In the majority of practical cases, a probe tone frequency of 226 Hz is applied. This is due to the fact that higher frequencies may lead to standing waves and the measurement result is thus influenced by the geometry of the auditory meatus. With higher probe tone frequencies (e.g. 678 or 1000 Hz), however, more information is obtained about the physical processes in the middle ear, possible pathological changes, and the effects of surgical intervention.

#### 4.1.1 Tympanometry

In the context of tympanometry, it is not the impedance itself but its reciprocal (the admittance) which is measured. Since the impedance describes the resistance of the middle ear, the admittance corresponds to the compliance of the middle ear apparatus to transmit the sound to the inner ear. A high compliance corresponds to a low impedance to sound waves and thus to a high mobility of the tympanic membrane. The most favorable vibration properties of the tympanic membrane are seen when the external pressure is equal to the pressure in the middle ear, which is generally the case for normal atmospheric pressure. Hence a regular tympanogram measured at 226 Hz has its compliance maximum at normal pressure (p=0) with a normal range of ±150 daPa (type A according to Jerger).

This compliance maximum is the most important feature of a 226 Hz tympanogram. It is quantitatively described by its position, its height, and a form parameter. The height of the summit varies enormously in different individuals. If the maximum compliance is significantly above the normal range (tympanogram type AD “deep” according to Jerger), a scarred slack tympanic membrane, an interruption of the ossicles, or a defect of the incus are probable. A very flat compliance summit (type AS “shallow”) indicates a scarred rigid tympanic membrane, otitis media, or beginning otosclerosis. The shift of the maximum to positive or negative values (type C) reveals positive or negative pressure in the tympanum. Overpressure may occur in case of tube dysfunction, negative pressure is observed in cases of oxygen-consuming inflammatory middle ear diseases, for example beginning otitis media without effusion. Variations of the middle ear pressure in a range of ±100 daPa have no diagnostic relevance [157]. If a viscous effusion is found in the tympanum, the tympanogram does not have a compliance summit, it is flat with a soft increase to the negative pressures (type B). Flat tympanograms may also indicate incorrect measurements (e.g. blocked probe tip or contact with the wall of the auditory canal). Tympanograms with more complex graphs may be seen especially for higher probe tone frequencies at the transition from negative to positive pressure; therefore, the measurement should always start with overpressure [158]. In this way, defects of the tympanic membrane as well as an incorrect position of the probe plug can be recognized.

Tympanograms that are measured with higher probe tone frequencies – usually 678 and 1000 Hz – might have more than one summit, symmetrically arranged around the middle axis (corresponding to normal atmospheric pressure) in absence of pathological processes [159]. The reason for the use of higher probe tone frequencies is that a high percentage of the tympanograms with 226 Hz is inconspicuous, especially in children, even in cases of known otitis media [160], [161]. The physical properties of mechanical systems are generally more sensitive for changes of the parameters of mass, friction, and elasticity near the resonance frequency that amounts to about 1 kHz for adult middle ears. Since the resonance frequency of pediatric middle ears is higher because of the smaller dimensions, an impedance measurement at low frequencies is less suitable to detect pathological changes. An extensive exploration includes measurement at several probe tone frequencies (multifrequency tympanometry) and the separated assessment of a real and imaginary part (multi-component tympanometry) of the impedance or admittance. Even in the context of those tympanograms that sometimes have more than one summit, changes of the middle ear pressure lead to a shift and fluid in the middle ear leads to flatter graphs [162]. Recently, different and partly promising paradigms on multi-frequency tympanometry and broadband reflectance were presented and tested [163], [164], [165], [166]. Up to now, however, none of the approaches has been established in practice. See Table 18 (Tab. 18).

#### 4.1.2 Stapedius reflex

The second field of application of impedance audiometry is the detection of the stapedius reflex. It is recorded via the registration of an increased impedance during acoustic stimulation of one ear but the reflex can also be elicited by tactile or electric stimulation. Impedance changes occur when strong acoustic stimuli cause the stapedius muscle inserting at the stapes (and possibly also the tensor tympani muscle that inserts at the handle of the malleus) to contract. This leads to a stiffening of the ossicular chain and thus to an increased impedance of the eardrum. Muscle contraction – and impedance change – follows stimulation after a short delay (latency of about 10 ms); it lasts for the duration of the stimulus and generally it decreases after about 10 s.

The reflex-like contraction of the stapedius muscle occurring in cases of loud sounds impedes vibrations of the stapes and thus protects the inner ear from too high sound intensities. The stapedius reflex is an acoustico-facial reflex, this means that the middle ear, inner ear, and hearing nerve belong to the triggering (afferent) branch; the efferent part is the motor facial nerve innervating the middle ear muscles. Afferent and efferent branch of the reflex arc are connected with each other in the nuclei of the hearing and facial nerves of the olivary complexes. Since the efferent branch of the middle ear muscles innervates both ears, monaural sound leads to bilateral impedance changes. The stapedius reflex can be triggered by ipsilateral and contralateral stimulation. In most practical cases, the question must be clarified if the reflex can be triggered in one ear, i.e. mainly the stimulated ear. However, there might also be questions that focus on the ear with the probe.

In order to register the ipsilateral stapedius reflex, a reflex triggering sound with a frequency selected by the examiner (500 Hz, 1 kHz, 2 kHz, or 4 kHz) and intensity (level between 70 and 110 dB HL) is issued to the probe ear for a limited time (1 s) in addition to the probe tone. For measurement of the contralateral stapedius reflex, a headphone is placed on the stimulated ear; on the probe side the impedance probe is placed in the auditory canal. Measurement of the reflex is always performed with the pressure with largest compliance (maximum of the tympanogram). Starting at about 70 dB HL, the reflex threshold is found by increasing the stimulus level. The reflex threshold is defined as the lowest stimulus level at which an increased impedance can be registered that is typical for the reflex. In normally hearing people it amounts to 70–90 dB HL, in hearing impaired people it might be higher. A low percentage of normally hearing people does not have this reflex.

Generally, the stapedius reflex is only triggered by stimuli that are characterized by high subjective loudness. The reflex threshold does not correlate with the level of the stimulus (dB HL) but rather with a level related to the individual hearing threshold of the examined ear (dB SL). In cases of middle ear hearing impairment, the reflex threshold is higher by the amount of the hearing loss. If the hearing loss is more than 30 dB, the reflex cannot be triggered because in this case a stimulation level of at least 110 dB HL (≥70 dB above the threshold) would be required. Therefore, the reflex cannot be triggered in an ear affected by middle ear hearing loss – and (because of other reasons) it cannot be registered in most of the cases. In case of inner ear hearing loss, the reflex threshold is constant up to a hearing loss of 50 dB and increases linearly with larger hearing loss. This means that in many sensory hearing impairments, the reflex threshold is closer to the hearing threshold than in normally hearing persons manifesting the narrow dynamics of the ear with hair cell damage (“Metz recruitment” if the difference of stapedius reflex and hearing threshold is ≤50 dB). Inner ear hearing impairment is only seen on the stimulus side but not on the probe side.

The influence of neural hearing loss on the stapedius reflex depends on the question whether the disorder is located peripherally of the reflex connection in the hearing nerve or more centrally in the brainstem. In the first case, the reflex threshold is clearly increased (with negative Metz recruitment: the difference between reflex threshold and hearing threshold is >50 dB) or a pathological reflex decay is observed or the reflex is completely missing; in the second case, the stapedius reflex is not impaired. In the stimulated ear as well as in the probe ear, conspicuous findings may be detected. Even in cases of supraclavicular paresis of the facial nerve as well as damages of the facial nerve that are located behind the branching of the stapedius nerve, the stapedius nerve can be generally registered; peripheral paresis of the facial nerve that usually does not influence the hearing threshold, however, causes failure in the probe ear. See Table 19 (Tab. 19).

### 4.2 Otoacoustic emissions

Sound waves that emerge from the inner ear and are transmitted via the ossicles and the eardrum into the external meatus, are called otoacoustic emissions (OAE). Spontaneous emissions (SOAE) which are present without acoustic stimulation are distinguished from evoked emissions (EOAE) which occur during or after acoustic stimulation. OAEs originate from non-linear and active processes of the cochlear sound pre-processing that manifest already in the micro-mechanics of the basilar membrane and are responsible for the high sensitivity, the large dynamic range, and the capacity of the ear to distinguish frequencies [3]. The source of cochlear emissions are microscopic movements of the outer hair cells (OHC). Specimens of cultured OHC can be triggered to active contractions by chemical, electric, and mechanical stimuli. In the context of the physiological hearing process, OHC perform stimulated contractions and elongations in a delimited area of the cochlea corresponding to the frequency of the stimulus. On the one hand, the maximum deflection of the basilar membrane is increased and narrowed and on the other hand, a secondary travelling wave of small amplitudes arises which spreads retrograde, radiates via the eardrum and leads to measurable variations of the sound pressure in the outer ear canal.

Evoked OAEs (EOAE) are classified into post-stimulatory (delayed) transient evoked otoacoustic emissions (TEOAE) and per-stimulatory emissions. The latter ones are further differentiated if the frequency of the emissions corresponds to the stimulus (stimulus frequency otoacoustic emissions, SFOAE) or if the frequency of stimulus and response are different (distortion product otoacoustic emissions, DPOAE). TEOAEs can be reliably measured, they are well established in audiology. In contrast, the registration of SFOAE is difficult so that among the per-stimulatory OAE exclusively the DPOAE are used for diagnostic purposes [167], [168].

TEOAE and DPOAE reflect two aspects of the same active and non-linear cochlear amplifier measured in different ways. The different measurement techniques have an impact on the practical application and the information because, with comparable stimulus levels, DPOAE can be measured with a higher sensitivity than TEOAE, i.e. also in the context of severer hearing impairment [169].

#### 4.2.1 Transient evoked otoacoustic emissions

For the measurement of TEOAEs, the acoustic signal is registered immediately after a transient and repeated stimulation with click stimuli in the auditory canal. By means of shielding as well as with analogue and digital signal processing (selection and averaging of many signal components, elimination of linear signal components), the physiological OAE signal is separated from ubiquitous disturbances.

The result of TEOAE measurement is displayed as graph that reflects the average time-dependent course of the post-stimulatory sound pressure in the auditory meatus. The transformation of these graphs in the frequency ranges provides the spectra of TEOAE and residual noise. Furthermore, a correlation coefficient (reproducibility) is calculated and the variance of the residual noise is estimated. If the reproducibility is higher than 60%, TEOAE are considered as being proven [170], [171], [172]. This criterion corresponds to the requirement of a signal to noise ratio of at least 6 dB [173].

The most difficult part of the examination is successfully performed and the most important part of the information contained in TEOAE is used with the reliable and clear differentiation between signals of cochlear origin and noise interference. With regard to basic clinical use, OAE behave like a binary variable or a dichotomous system with only the two states: “present” and “not present”. An ear where delayed emissions can be measured, has a nearly normal hearing threshold at least in part of the audiogram. The incidence of measurable TEOAE gradually decreases with increasing inner ear hearing loss from 100% down to 0%. To a small extent, the details of this transition depend on the intensity of the stimulus; for a level of L=80 dB peSPL, the 50% incidence corresponds approximately to the hearing loss of 30 dB HL [174], [175], [176]. Hereby the lowest hearing loss , i.e. the most favorable hearing threshold, occurring in the frequency range of 1–4 kHz, is relevant, because already a locally delimited normal inner ear function is sufficient for the appearance of TEOAE. Areas with more significant functional deficits do not contribute to the cochlear response. The evaluation of the frequency spectrum and the reproducibility of the emissions calculated for the single frequency bands (or the signal to noise ratio) allows a delimitation of the frequency range affected by the hearing loss.

The aforementioned correlations between hearing threshold and TEOAE refer to the special case of merely sensory (endocochlear) hearing disorders. Of course, also conductive hearing loss has an effect on the measurement and the amplitude of TEOAE. The impaired sound transmission of the middle ear apparatus leads to a damping of stimulus and emission. If the middle ear component is strong enough (20 dB or more) and if all frequencies are affected, no TEOAE can be measured. In contrast, a merely retrocochlear hearing loss has no influence on the TEOAE. It is characteristic for some neural hearing disorders to find the constellation of poor hearing threshold with nearly normal emissions [177], [178], [179].

Measurement of TEOAEs does not allow for quantitative and frequency-specific determination of the hearing threshold. The spectrum of each delayed emission has a fine structure of irregular and individually different peaks and notches, but they do not correspond to hearing loss in the tone audiogram [180], [181]. A correlation between TEOAE spectrum and audiogram can only be shown statistically but not in individual cases [182]. Only if no emissions occur in a very broad frequency interval, it may be interpreted as hint to an elevated hearing threshold for those frequencies. Another particularity that has to be taken into consideration for isolated hearing loss of low frequencies is described in further publications [170], [171]. 

When a broadband stimulus (click) is used, the spectrum of the TEOAE contains all stimulus frequencies for which the cochlea has a nearly undisturbed function. A hearing loss that exceeds the limit of 30 dB leads to disappearing of the delayed emissions. The complete absence of a physiological “echo” means that the hearing loss exceeds this value at all frequencies. On the other hand, the presence of reproducible emissions allows the conclusion that at least part of the hair cells have a nearly normal function and the “minimal hearing loss” (most favorable value in the audiogram) is less than 30 dB. The validity of these statements is limited to the frequency range between 1 and 4 kHz, hearing losses outside this region cannot be assessed with TEOAE. 

The dichotomous nature in combination with the sensibility limit of 30 dB hearing loss qualifies TEOAE as ideal procedure for the early detection of congenital hearing disorders in the context of newborn hearing screening [183], [184], [185], [186], [187], [188], [189], [190], [191], [192], [193], [194], [195], [196], [197]. The measurement and evaluation procedures that were automated for this purpose use a more sophisticated signal detection procedure based on statistical analysis [198], [199]. The measurement of TEOAE plays a central role also within the confirmation diagnostics after conspicuous screening (follow-up) [200].

Besides dichotomy, the TEOAE are a source of differentiated and also diagnostically valuable information if their amplitude and its behavior is considered after repeated measurements or after changing the examination conditions. An application of this kind is the contralateral suppression of TEOAE that is transferred by the efferent innervation of the OHC via the medial olivo-cochlear bundle [201], [202], [203], [204], [205]. This suppression can be identified by measurement of the click-evoked TEOAE when providing sound to the other ear with a broadband noise that has a level of 50–70 dB HL. In cases of auditory synaptopathy/neuropathy (AS/AN), the suppression effect is lower or completely missing [162], [206], [207]. In order to prove this effect, the "nonlinear" stimulus sequence, which eliminates the linear components of the response, should not be selected (“linear mode”). A rapid change between the recordings with and without contralateral sound (“Lyon mode” according to [208] in the measurement system of ILO88) favors the evidence that is difficult to provide because the residual noise generally increases when the difference between two signals contaminated with noise is taken. The suppression effect is considered as missing when the amplitudes measured with and without contralateral masking differ by less than 1 dB.

The comparison between two TEOAE measurements that were carried out under the same conditions at different times contains the potential of objectifying changes of the hair cell function. It could be revealed that a change of the (noise corrected) emission amplitude is significant when the difference of the amplitudes is 4 dB or more [209], [210]. Monitoring of the TEOAE after sudden hearing loss shows parallels between increasing amplitudes and hearing improvement in many but not all cases [211]. According to some reports, the TEOAE are appropriate for early detection of noise induced hearing loss in the inner ear [212] and for the sensitive detection of possibly even subclinical effects of medication with ototoxic substances on the hearing capacity [213]. See Table 20 (Tab. 20).

#### 4.2.2 Otoacoustic distortion products

The non-linearity of cochlear signal processing leads to the fact that the inner ear cannot process two close frequencies independently. If the inner ear is stimulated with a mixture of two pure tones, the physiological processing results in secondary tones with frequencies that are not contained in the stimulus. Those distortions can be measured as DPOAE (distortion product otoacoustic emissions) in the auditory meatus [214].

The distortion products are evoked by a stimulus that is composed of two supra-threshold sinus tones of nearly equal intensity with the frequencies f1 and f2=1.2xf1 (e.g. f1=4.0 kHz and f2=4.8 kHz). During the DPOAE measurement, the amplitude belonging to the frequency 2f1-f2 of the distortion product is extracted from the spectrum of the microphone signals, which is increasingly freed from background noise in the course of the averaging process, and displayed at the end of the measurement series for several stimulus frequencies together with the amplitude of the respective background noise of a narrow frequency band (DP-gram). For reconstruction of the stable physiological signal from the stochastic noise, the general rules are applied according to which the quality of the result, described by the signal to noise ratio (SNR), doubles only after a fourfold increase of the measurement time. It is due to the narrowband registration that DPOAE measurement achieves extremely good SNR values (more than 100 standard deviations of the amplitude of the residual noise are regular). This is the reason why in the context of advanced hair cell damage, the vitality signs of a small residual population of OHC can still be identified in contrast to TEOAE [169]. 

Regarding the correlation between DPOAE and the extent of sensory hearing loss, the rules are qualitatively very similar to those for the TEOAE:

- The DPOAE evoked by the frequencies f1 and f2=1.2xf1 occur with an incidence of nearly 100% in normally hearing ears [215].
- The emission amplitude decreases with increasing hearing loss.
- If the hearing loss at the frequency f2 exceeds 50 dB, no DPOAE can be identified in the statistical average [174].
- Incidence and amplitude of DPOAE are not influenced by a pure retrocochlear hearing impairment.
- Conductive hearing losses first cause a reduction of the DPOAE of lower frequencies, in cases of severer hearing loss, the DPOAE disappear completely.

These statements are valid for all stimulus tone pairs for which the frequency of f2 is between 1 and 4 kHz. For lower frequencies, the incidence of measurable emissions is clearly below 100% even in normally hearing ears; for higher frequencies, the DPOAE measurement is often falsified because of technically induced distortions. 

Due to their properties (automatic measurement and evaluation, dichotomy, sensitivity for primarily damaged structures in the context of innate hearing disorders), DPOAE turned out to be appropriate for newborn hearing screening. The application in this context has been reported several times [192], [216], [217]. In existing screening programs, however, DPOAE play only a minor role because the screening result may be inconspicuous up to a hearing loss of 50 dB, but in the follow-up they are a well-defined component of pedaudiological examination in order to exactly differentiate the hearing impairment [200].

Beyond dichotomous statements, DPOAE achieve quantitative results that are expressed for example in approaches to frequency-specific objective determination of hearing thresholds. One of the approaches described in the literature [218], [219], [220] focuses on the question whether the hearing threshold can be measured in a frequency-specific way and quantitatively by means of the dependency of the stimulus level of the DPOAE amplitude (“DP growth function”). The method was not established in practical audiometry, because, among other reasons, its application is generally limited to low-grade hearing loss. Furthermore, the frequency specificity of DPOAE, which had been overestimated initially, does not significantly exceed the one of TEOAE [182]. Also the possibility to differentiate between conductive and sensory hearing loss based on the DP growth function [221], [222] could not enter into clinical practice. The method is also not able to differentiate a vestibular schwannoma from merely cochlear hearing disorder because already small vestibular schwannomas are accompanied by cochlear deterioration [69].

Comparable to TEOAE, also DPOAE were investigated regarding the contralateral suppression of the emission amplitude triggered by efferences of the hearing pathway, [223], [224], [225], [226], [227]. In specialized, especially pedaudiological centers the procedure is applied in order to obtain an objective correlate of complex peripheral-neural (AN/AS) or central (CAPD) hearing disorders [205], [228]. 

As a direct image of non-linear sound processing in the inner ear, DPOAE are directly related to the functionality of the OHC and thus generally suitable to objectively observe damage or recovery of the hair cells and ideally even more sensitively than tone audiometry. Their advantage in comparison to TEOAE, which are limited to non-linear signal components because of the special stimulus paradigm, is the assessment of the complete undistorted physiological signal. It has been suggested to apply DPOAE for follow-up and possibly early detection of beginning noise-induced or ototoxic damage [212], [229], [230], [231], [232], [233], [234]. In occupational medicine, the monitoring of DPOAE as well as TEOAE is useful for employees working in noise, as OAE measurement is also recommended for medical reports on noise-induced hearing loss [233], [235] based on the Königstein recommendations [99]. DPOAE also reflect the influence of ageing on the hair cell function and thus the development of age-related hearing loss [236], [237], [238], [239], [240] although the sensitivity is not higher than the one of the pure tone audiogram. See Table 21 (Tab. 21).

### 4.3 Auditory evoked potentials

Auditory evoked potentials (AEP) are electric voltages of physiological origin that can be triggered by acoustic stimuli and measured by means of electrodes. The sum of all procedures for the examination of hearing characteristics on the basis of AEP is called electric response audiometry (ERA) [241]. Those are objective functional tests that allow a quantitative determination of the hearing threshold. Already because of this property, ERA plays a major role among audiometric tests. Special benefit is obtained by the fact that ERA may yield differential diagnostic or topodiagnostic insights, in particular for the differentiation between sensory and neural hearing disorders.

The difficulty of measuring AEP is that the signal to be detected (the AEP) has a very small amplitude and is strongly contaminated with interfering noise (spontaneous EEG). The amplitude of the interferences – especially when muscular potentials and external electromagnetic signals are present additionally to the EEG – may be significantly above the amplitude of the target signal (depending on the frequency). The measurement of AEP requires first a substantial avoidance of interferences (electric shielding), the relaxed and comfortable positioning of the patient, a linear EEG amplification with high common-mode rejection, filtering of the EEG signal, rejection of artefacts, and effective improvement of the signal to noise ratio by signal averaging. The optimization of these procedures leads to a higher quality and reliability of the results [242]. The only relevant measure for quality and accuracy of the final results is the effective amplitude of the residual noise, which should be part of the documentation according to DIN EN-60645-7 [243]. Stimulus responses of small amplitudes (i.e. in particular near the threshold) cannot be reliably identified in recordings with high residual noise. The residual noise also determines the accuracy of the parameters derived from the primary curves [244].

A major part of electric reactions of the hearing system can only be registered as delayed and transient response at the beginning of a time-limited stimulus. The averaging of many stimulus-related signal epochs (sweeps) results in a time-dependent curve that contains the EEG noise reduced in amplitude (residual noise) in addition to the AEP. The diagnostic statements regarding hearing are based on the evaluation of these curves measured with stimuli of different quality (e.g. frequency or time course) and intensity and the parameters resulting from a subsequent curve analysis (latency, amplitudes, side differences).

For the generation of measurable evoked potentials, many nerve action potentials have to be triggered with a high degree of synchronization. This can be achieved only with stimuli with rapid inherent changes of some of their properties. Most frequently, this property is the intensity or the sound pressure of the stimulus. The most drastic changes are accomplished by turning the stimulus on and off. The usual transient stimuli are the broadband click and the frequency-selective tone pulse. Since high frequency selectivity and short duration of stimulus exclude each other, responses that require a high degree of synchronization provide only very limited frequency-specific information. On the other hand, the frequency-specific stimulation yields only very rough information about the latency of the potentials.

The transient AEP are composed of many single voltage peaks which originate from different parts of the ascending hearing pathway and may be classified in 3 groups according to their latency. Early auditory evoked potentials (EAEP) describe the components in the time range of 1–10 ms, followed by middle auditory evoked potentials (MAEP) with latencies up to 50 ms, and slow AEP (SAEP) with latencies up to 500 ms. The associated methods are BERA (brainstem electric response audiometry) for early, MLRA (middle latency response audiometry) for middle, and CERA (cortical electric response audiometry) for the slow AEP. This notation roughly mirrors the anatomical site of the generators or sources of the responses. The classification is not standardized, but it may be considered as certain that the “wave” J1 is generated in the hearing nerve, EAEP J3 and J5 in the brainstem, MAEP in the thalamus and the primary auditory cortex, and SAEP in the auditory cortex.

Generally, the normal values for latencies, latency differences, and amplitudes of the responses which are essential for the identification of abnormalities are device-specific because of their dependence from the details of stimulation and signal processing. Thus the values taken from tables are not universally valid [245]. According to the international standard of DIN EN 60645-7, the manufacturer is responsible to deliver normal values making reference to their source [243]. 

#### 4.3.1 Electrocochleography

At the time of its development, the transtympanic electrocochleography (ECochG) played a major role among the objective methods for hearing measurements [246], [247]. The advantage of this method is that it allows the registration of very early AEP with only low deterioration by disturbances. Hence, sensorineural activity of pre- and postsynaptic processes in the inner ear can be displayed. Nowadays, the practical application of ECochG is limited to differentiated diagnostics in children who are suspected to have high-degree hearing loss [162], [248]. The examination for which the tip of a needle electrode is placed on the promontory through the eardrum, is performed under general anesthesia in most cases. Via the tube of an insert earphone, the acoustic stimulus is transmitted into the auditory canal.

The result of ECochG is composed of cochlear microphonics (CM), the compound action potential (CAP), and the summation potential (SP). These components can best be described when stimulation is performed with frequency-selective tone pulses. The CM is an oscillation following the frequency, phase, and duration of the stimulus; it reflects the receptor potential of the sensory cells. The CAP corresponds to the sum of action potentials of all stimulated hearing nerve fibers and is displayed as negative deflection of a latency depending on the stimulus level of about 2–7 ms. The SP appears as a vertex-positive DC voltage component, the duration of which is the same as the stimulus pulse; it reflects the voltage difference between scala media and scala tympani. Since the presynaptic CM is locked to the phase of the stimulus whereas the postsynaptic CAP does not follow the phase, both components may be isolated by the separate recording of the responses following condensation and rarefaction stimuli and the subsequent calculation of sum and difference curves.

The determination of the threshold based on the CAP is much more accurate than that obtained from BERA and is frequency-specific in the range from 500 to 4000 Hz. Empirical data show that the “true” (behavioral) hearing threshold is only a few dB lower than the stimulus response threshold of the CAP [162]. Regarding the frequency-specific objectification of the threshold in children, ECochG is more powerful than most other methods. Beyond the determination of threshold, ECochG contributes to the diagnostics of AS/AN; the characteristic finding is the constellation of clearly pronounced CMs and depleted or lacking CAPs [162], [206], [207], [248], [249].

In the context of differential diagnosis, ECochG may further contribute to the detection of endolymphatic hydrops in the diagnosis of Menière’s disease. The component SP reflects the voltage differences between scala media and scala tympani, its polarity and amplitude are determined by the movement of the basilar membrane, especially with regard to non-linearity and asymmetry. Therefore, an increased SP can be expected when the basilar membrane is displaced from its balanced position due to disturbed water balance [250], [251], [252]. However, not the absolute amplitude of the SP but the relation between the amplitudes of SP and CAP is enlarged in comparison to control groups [253]. The consideration of the quotient SP/CAP could not be considered as the basis for a sharp criterion but rather as a “thumb rule” with many exceptions. A meta-analysis revealed an amplitude relation of 0.42 as critical value for differentiating between hydrophic and normal ears [254]. See Table 22 (Tab. 22).

#### 4.3.2 Early auditory evoked potentials

With the BERA, all early auditory evoked potentials (EAEP) from hearing nerve and brain stem are examined. The “brainstem audiometry” analyzes the timeframe from of 1 to about 12 ms following the stimulus. EAEPs can only be measured with sufficiently high amplitudes if the stimulus generates a high degree of neural synchronization, as it is ideally the case with a click stimulus. The click is not suitable for the frequency-specific determination of the hearing threshold, however, there is the alternative of stimulating with pulses or chirps (see below). The early potentials are very stable with respect to vigilance variations and pharmacological influences so that their measurement is also possible during sleep or under anesthesia. Regularly, they are present at birth, however, during the first months of life they are observed in a modification that differs from the adult pattern regarding morphology and latency times due to the immature hearing pathway. The EAEPs consist of several components of peaks or “waves”, called J1 to J5 or waves I to V, which are generated in different places of the ascending hearing pathway from the hearing nerve up to the brainstem.

The complete wave pattern can only be observed if high supra-threshold stimuli are applied. The amplitudes of the responses increase with growing stimulus intensity, whereas their latency times decrease. These relations are displayed in diagrams (e.g. “latency-intensity-diagram) which facilitates the recognition of abnormalities [255]. The most important fields of application of BERA are the objective determination of the hearing threshold in infants, toddlers, and children as well as the diagnostic differentiation between sensory and neural hearing disorders.

##### 4.3.2.1 Definition of the hearing threshold by means of click-BERA

The “objective” quantity deduced from the recordings corresponding to the behavioral hearing threshold is the response threshold. This objective threshold is defined as the lowest stimulation level at which a reproducible physiological response can be recognized visually from the curves or in case of sufficient quality, extracted from the input-output function of wave V. Since this wave can usually be detected down to the subjective hearing threshold (with deviations amounting to 15 dB or less) with click-evoked EAEP, the hearing threshold in the high frequency range (about 2–4 kHz) can be estimated reliably and with acceptable accuracy.

Different hearing disorders lead to characteristic changes of the EAEP which in most cases allow differential diagnostic statements based on click-BERA and they are the basis for objective estimation of the hearing threshold. In the case of conductive hearing loss, the effective stimulus level is reduced by the difference between the threshold for bone and air conduction. This level reduction leads to longer latencies and lower amplitudes of the EAEP. The response threshold is elevated and all level-latency and level-amplitude curves are horizontally shifted by the amount of the sound conduction component [255]. The interpeak latencies remain unchanged. In cases of malformations of the outer ear, where the stimulation via air conduction is difficult or impossible, as well as in cases of combined hearing loss, the BERA should also be performed with bone conduction. In this way, sensory hearing loss and its extent can be measured and differentiated from conductive hearing loss [256], [257], [258].

Another pathological factor that affects the morphology of brainstem potentials is the recruitment. In the supra-threshold range, the amplitudes of the EAEP of patients suffering from inner ear diseases behave similarly as the steep subjective loudness growth. Close to threshold, the potentials are delayed, in the supra-threshold region, the latencies are nearly regular. Inbetween, a steep amplitude growth can be observed. The latencies of the potentials may be essentially prolonged near threshold, with increasing stimulus level they approach the normal values [255]. Especially in case of basocochlear hearing loss, different latency courses and altered curve morphologies may result that do not allow a general definition of expectation ranges for the recognition of potentials. As a consequence, the evaluation of BERA findings may be difficult not only for algorithms of automatic wave recognition but also for experienced examiners.

For high frequency cochlear hearing losses, the response threshold is closely related to the hearing threshold in the frequency range between 2 and 4 kHz [259], [260], [261], [262]. In many cases, the fast EAEP components are missing and the wave J1 is delayed so that the central conduction time (defined as the latency difference t5-t1) is reduced. The severer the hearing loss is, the shorter is the interpeak latency [263]. Moreover, the difference between the response threshold and the pure tone hearing threshold (defined as mean value at 1 kHz, 2 kHz, and 4 kHz) can be substantially enlarged with increasing hearing loss: for 25 dB hearing loss the response appears at 40 dB HL and for 60 dB hearing loss the response threshold can be expected to be at 100 dB HL [245].

Due to the acoustic properties of the click stimulus on the one hand and the biomechanics of the cochlear on the other hand, a particular low frequency hearing loss does not influence the click-evoked EAEP and so it cannot be explicitly detected by means of click-BERA.

##### 4.3.2.2 Frequency-specific BERA

The lack of frequency specificity of the click-evoked EAEP limits a wide application despite their otherwise favorable properties. This was the motivation for intensive research efforts regarding frequency specific audiometry by means of early potentials. One of the numerous approaches is based on stimulation with short tone bursts and simultaneous masking with notched-filtered broadband noise (notched-noise-BERA). The responses obtained with this procedure can generally be measured for frequencies between 500 Hz and 4 kHz – especially for the test frequencies of 2 and 4 kHz – nearly down to the hearing threshold [264], [265], [266]. According to the properties of the cochlear travelling wave, the latency times of the responses are longer for lower than for higher frequencies. The same physiological mechanisms have the effect that the neuronal activity at low frequencies is less well synchronized and this limits the accuracy of the derived hearing thresholds. In particular, the results obtained at 500 Hz must be interpreted with caution in the context of therapeutic decisions. The experience from clinical routine shows that the threshold differences in this frequency range may amount to 40 dB and more. Since the threshold must be assessed 4 times per ear (for each frequency), the duration of the examination is long, which is another disadvantage of notched-noise BERA. Furthermore, much experience is required in order to unequivocally identify the response at low levels near threshold.

In the diagnostic use of the FAEP, the general incompatibility of temporal (click) and spectral sharpness (tone pulse) is associated with fundamental difficulties: Apparently, the neural response is either well-synchronized and thus easily measurable, or it is frequency-specific and temporally dispersed. A useful synthesis of these two extremes seems to be possible with the chirp. For the design of this short-term stimulus, a wave form composed from a frequency continuum of defined bandwidth. Phases, amplitudes, and envelope of the single components are selected on the basis of either experimental [267], [268], [269], [270], [271] or of model calculations [272] in order to compensate the dispersion of the basilar membrane [273], [274], [275], [276], [277], [278]. Thus, a large section of the basilar membrane is stimulated at the same time. This stimulation is synchronous but still wideband. To improve the frequency selectivity, sections from the wideband chirp transformed into the frequency domain are isolated and re-transformed into a low, medium and high chirp [279], [280], [281], [282]. In order to make sure that with these sections only a defined frequency area of the cochlear is stimulated, it is appropriate to apply this stimulus together with an a masking noise – comparable to notched-noise BERA [280], [281], [283]. The spectral properties of the chirp are similar to the tone pulse. Due to the high degree of synchronization, however, the AEP amplitudes are larger at identical stimulus levels and thus the threshold transition is easier to detect [284], [285].

##### 4.3.2.3 Differential diagnostics of hearing loss with BERA

In order to obtain differential diagnostic statements, EAEP are measured with click BERA. Latencies and amplitudes are determined for all stimulus levels. By comparing the latency-intensity-diagrams with the normal reference curves, the calculation of latency differences, especially the cochlea-mesencephalic latency difference t5–t1, and the evaluation of side differences, conductive, sensory, and neural lesions can be distinguished. Conductive hearing impairment is characterized by prolongation of the latencies of all waves because lower stimulus levels are associated with longer latencies. Due to the constant attenuation of all stimuli, the latency and amplitude functions are shifted along the level axis. In case of inner ear hearing loss – depending on the frequency range affected by the disorder – latency prolongations occur only for stimuli close to the threshold while often normal latencies are observed at high stimulus levels. The effect of a neural hearing disorder is a prolongation of the neural processing time which is independent from the stimulus level; the latency intensity function is therefore shifted along the latency axis, the amplitude-intensity function is typically flat, i.e. the amplitude does not grow with increasing stimulus level.

For a long period, especially as long as MRI scans with sufficient image quality were not yet available, the registration of EAEP was part of the standard audiological inventory, with the expection of additional specific information about retrocochlear disorders. The hit rate is very high for advanced tumors [286], whereas different trials could show that EAEPs are less sensitive in case of small tumors. Especially vestibular schwannomas with a diameter of less than 10 mm are difficult to diagnose, with a sensitivity below 60% [287], [288], [289], [290], [291], [292], [293].

The diagnostic criteria for vestibular schwannoma are the latency of the wave V, the prolongation of the interpeak latency t5–t1 and the corresponding interaural differences. Pathophysiologically, the latency prolongations are supposed to originate from the tumor-related impact on the hearing nerve fibers and the loss of neural synchronization [294]. The critical limits reported in literature are far from being uniform; however, the diagnosis can be regarded as confirmed if the interaural differences of the latency t5 exceeds the limit of 0.3 ms [245], [295], [296], [297]. Sensitivity and specificity of the procedure depend strongly on the limiting criterion: rigorous values increase the sensitivity and reduce the specificity – and vice versa. An approach to increase the efficiency of BERA especially for detection of small vestibular schwannomas was described by Don et al. (1997) [298]. If the click stimulus is presented together with a high-pass filtered pink noise of different threshold frequencies, frequency-specific responses – the so-called "stacked ABR", which originate in different cochlear areas – can be generated as a representative of the synchronous neural activity [298], [299], [300].

Under the supposition that the high-frequent hearing nerve fibers which are crucial for the stimulus response to unmasked clicks, are not affected by small tumors, the pathological latency changes of the low-frequency “stacked ABR” as well as the associated amplitude reduction of the total response may serve as tumor indicator. With this procedure, the sensitivity may be increased to 95% and the specificity to 88% [298], [300]. The “stacked ABR” technique, however, is rather complex and rarely available in clinical equipment.

Another possibility to improve the effectivity of BERA in the diagnosis of small vestibular schwannomas was suggested recently. If the registration of EAEPs is extended down to 40 dB HL in patients with low-grade hearing loss, the consideration of the combination of “pathological absolute latency of the wave V” and “pathological interaural difference of the wave V” can improve the detection of tumors larger than 5 mm [301].

##### 4.3.2.4 Newborn hearing screening with automatic BERA

EAEP play a major role in the context of newborn hearing screening [302]. In comparison to OAE, their measurement requires higher efforts (placing electrodes, longer duration of measurement), but the results are more reliable regarding their relevance and the method also addresses pure neural hearing disorders. In a certain sense, OAE and EAEP are complementary regarding the condition of the child to be examined: motoric agitation of the extremities, for example, does not impair the registration of OAE but EAEP measurement becomes impossible; acoustic restlessness as e.g. noisy breathing, however, impedes OAE measurement but does not influence the quality of EAEP. This suggests the idea of a simultaneous registration of both signals [303], a method that has not been extended beyond the laboratory stage. In most screening programs, TEOAE and EAEP are combined in a two-stage protocol [162], [186]. The applied aABR (automated auditory brainstem responses) are different from conventional BERA in the sense that measurement and signal detection are automated. The algorithm used in this context is very similar to the one applied to TEOAE recordings [186], [192], [195], [302], [304], [305], [306]. See Table 23 (Tab. 23).

#### 4.3.3 Slow auditory evoked potentials

The slow or late cortical potentials SAEP assessed by means of CERA appear in the time interval of 100–500 ms post stimulus. The maxima and minima of the responses at about 100, 200, and 300 ms are classified as N1, P2, and N2. The amplitude of the response complex increases with growing stimulus level, the latency of the single components decreases with increasing stimulus level, but only near threshold. Morphology, latency, and amplitude of the stimulus responses may change enormously in sleep and vigilance variations. Reliable results can only be expected in wake and attentive patients. The maturation of the SAEP potential complex N1-P2 with respect to morphology, latency and amplitude is completed not earlier than with the end of the 10^th^ year of life [307]. Thus, the application of CERA in infants, children, and adolescents is limited: well-defined responses and a clear threshold transition may be considered as reliable results, missing responses, however, do not confirm deafness.

Responses of the auditory cortex can be evoked with nearly every acoustic stimulus of limited duration if the preconditions are fulfilled. Typically, pure tone pulses of defined frequencies are applied with a duration of 30–500 ms. If the level of these stimuli is modified, the response threshold can be determined for each frequency, yielding an objective pure tone audiogram. The accuracy amounts to about 10 dB. Since the conscious perception does not correspond to the activity in the primary auditory cortex but to the one in the higher association centers, the subjective hearing process cannot be measured with the SAEP. For this purpose, other examination procedures based on even later responses and partly requiring an active cooperation of the patient (event related potentials, ERP) are suitable. Although these cognitive responses, the most known being the mismatch negativity (MMN), can be recorded with some of the current commercial devices, they are applied rather in clinics and in specialized labs than in practices. They allow the examination of cognitive processes such as the differentiation between speech-related and non-speech-related stimuli or the discrimination of phonemes [241], [308], [309].

In the latency range of the classic SAEP, the observation of the component P50 in the time course of CI rehabilitation of single individuals led to scientific findings of high practical relevance [310], [311], [312], [313]. By demonstrating that the latency of P50 enters the corridor of the latency development of normally hearing children only in case of early provision, remaining irreversibly prolonged otherwise, scientific evidence is given for the limited cortical plasticity within the sensitive developmental phases. See Table 24 (Tab. 24).

#### 4.3.4 Auditory steady state responses.

While the measurement of the transient potentials dealt with up to now requires a pause between the acoustic stimuli, the so-called auditory steady state responses (ASSR) allow the examination of the hearing system in its steady state. The ASSR were first observed by Galambos et al. (1981) [314]. The acoustic stimulus is present without interruption while the EEG is registered and analyzed. Since the per-stimulatory analysis of the EEG signal requires another procedure, the measurements of ASSR are basically different from conventional ERA. By averaging or statistical evaluation, no time-dependent curve is reconstructed but a characteristic property of the EEG signal is elaborated that is associated with the physiological processing of the stimulus. Constant stimuli without any change in time, however, are not suitable for this purpose because they lead to stochastic and stationary neuronal activity, which cannot be separated from the EEG background.

Among several alternatives of ASSR, the responses on amplitude-modulated stimuli, which were first called “amplitude modulation following responses” (AMFR) by Kuwada et al. (1986) [315], are believed to be highly relevant since many years [241], [316], [317], [318]. For their detection, the amplitude of a continuous tone with a carrier frequency of 250–8000 Hz is modulated with a modulation frequency between 40 and 120 Hz [319]. The modulated stimulus evokes a synchronized activity of groups of neurons, probably in the segments of the hearing pathway that are located between the midbrain and the thalamus. Thus the modulation frequency is reflected in the EEG signal which is measured either laterally (vertex against mastoid) or medially (vertex against occiput). For the detection of this frequency, the amplified and narrowband filtered electrode signal is transformed into the frequency range and analyzed in the region of the modulation frequency with regard to amplitude and phase. The neuronal stimulus response is considered as being confirmed when the amplitude of the modulation frequency significantly sticks out from the floor of the averaged spectrum according to statistical tests or when the phase of this frequency related to the modulation of the stimulus deviates significantly from random distribution.

The impact of vigilance and maturation on the amplitude of the ASSR is generally stronger for high than for low modulation frequencies [319], [320], [321], [322], [323], [324]. The method claims to allow a frequency-specific, objective, and automatic determination of the hearing threshold in children. Some time ago, modulation frequencies in the range of 90 Hz and above were considered more suitable for the examination of children because the evoked EEG activity is less dependent on vigilance and maturation, compared to the responses of lower modulation frequencies [319], [320], [321], [322], [323], [324]; especially since the introduction of narrowband chirp stimuli of different stimulus rates [278], [325] this seems to be questionable in the light of recent studies [326], [327], [328], [329]. Even at low frequencies, an acceptable estimation of the hearing threshold can be achieved by means of 40 Hz ASSR in adults [285] as well as in children [327]. Since the identification of ASSR (which that may be considered as general term for those paradigms that consist of signal description and signal confirmation in the frequency range [330], [331]) is based on the modulation frequencies of the stimulus represented in the EEG, the stimulation can be performed with several differently modulated carrier frequencies and even in both ears at the same time as long as the stimulus levels exclude mutual masking [317], [318], [332], [333]. The same principle can be adopted for the application of chirp stimuli, where the repetition rates instead of the modulation frequencies are used for identification of the responses [327], [334]. See Table 25 (Tab. 25).

## Conclusion and outlook

The power of the current functional audiological diagnostics is based on the multitude of methods and their interrelation. If several tests are available to answer one diagnostic question, the results obtained for a target parameter will in general, but not always be congruent. In cases of contradictory results, the redundancy of the test inventory assures the possibility of discovering and eliminating inconsistencies. 

Another valuable characteristic of audiometry is the availability of numerous methods for objectifying findings. This option is missing in many other areas of medicine and would be very welcome there, too. In the field of audiometry it is always essential wherever there is no sufficient ability or readiness for cooperation of the patient which is urgently required for hearing tests. Beside pediatric audiology, also the ENT-specific reporting of expert opinion regarding hearing disorders is based on objective procedures. In those areas, as well as in occupational medicine [31] and in the context of prevention and early detection of noise-induced hearing loss according to the examination protocol G20 [335], the diversity of audiometric test procedures and their competent application are very efficient.

Despite the fact that the general situation may be regarded as definitely positive, there are still some gaps in the method inventory. For example, the recruitment, which cannot be ascertained subjectively in a reliable manner, cannot be objectified up to now. Moreover, a test for the identification of the vulnerable inner ear has not been found yet. Finally, the question of the electrical stimulability of the auditory nerve, which is fundamental before the decision to CI provision, can only be assessed by subjective methods and then only unreliably.

From the authors’ point of view, those are some of the future focuses of audiological functional diagnostics. Furthermore, procedures to determine the frequency selectivity (psycho-acoustic tuning curves, DPOAE suppression tuning curves) will gain in importance. It is known that the ability to distinguish frequencies is reduced in cases of noise-induced and other inner ear damage [336]. Possibly, the interest for this deficit will be enhanced by the increasing availability of frequency reduction procedures implemented in many new technical hearing aids. Up to now there is no test that can be applied in clinics or practices that might examine those effects.

The already mentioned pathological loudness growth merits deeper consideration. The opinion that the supra-threshold audiometry has been replaced by impedance measurement, BERA, OAE, and imaging procedures, has been repeatedly expressed and is meanwhile generally accepted. However, the mentioned alternatives are not able to discover a pathologically reduced dynamic range, which is crucial for the provision of hearing aids, despite their doubtlessly useful differential diagnostic power. The elimination of the SISI test with the argument that a negative SISI test is not always an indicator for nerve-induced hearing loss [99] only confirms a false expectation. A recruitment test can neither confirm nor exclude the presence of a tumor. However, if the argument is the lacking exactness of the SISI test, the consequence would be a more intensive use of loudness scaling.

Since several years, and still today, speech audiometry is intensively developed. Convincing studies [120], [121] indicate that the Freiburg speech intelligibility test which consists inherently of two speech tests, might be replaced by the WaKo test and GÖSA. Without any doubt, there is an acute need of extension beyond the Freiburg test for the assessment of speech intelligibility in noise. Possibly, this will result in its elimination. With regard to the “old” test material, recently the idea came up that speech audiometry can never be up-to-date because of the plasticity of living languages [100]. A continuous adaptation to the changes of daily speech is generally desirable but is not in accordance with the claim of standardization.

In objective audiometry, it becomes obvious that the consideration of the accuracy of results expressed in the residual noise will be better supported by appropriate equipment based on the standards (DIN EN 60645-6 [337] and DIN EN 60645-7 [243]). The difficulties in signal detection near the threshold are not a general shortcoming of the method, but they are due to the nature of threshold. The competent understanding of the impact of residual noise and the consequent differentiation between response threshold and hearing threshold will contribute to the replacement of false thresholds by less accurate threshold with defined confidence intervals. Especially the exploration of thresholds at low frequencies in pediatric patients will benefit from this aspect. Regarding the question which method is most appropriate for this field of application, valuable knowledge could be gained in the past years [283], [285], [327].

Another field of audiometry that has not yet been fully explored, is the application of objective hearing tests in the context of cochlear implantation [96], [226], [303], [338], [339] and the provision with implantable active middle ear prostheses [340], [341]. The need of objective procedures occurs in all phases of the therapeutic chain, from preoperative diagnostics on the suitability and indication (objective promontory test) via the intraoperative control and optimization of the electrode position (eCAP threshold and spread of excitation, SOE) up to the postoperative follow-up (e-BERA) and success prognosis (e-CERA). The applications are not relevant for practices outside specialized centers. They are only mentioned here to demonstrate the possibilities of development and future perspectives of audiometry. It is far from being done!

## Notes

### Competing interests

The authors declare that they have no competing interests.

## Figures and Tables

**Table 1:**
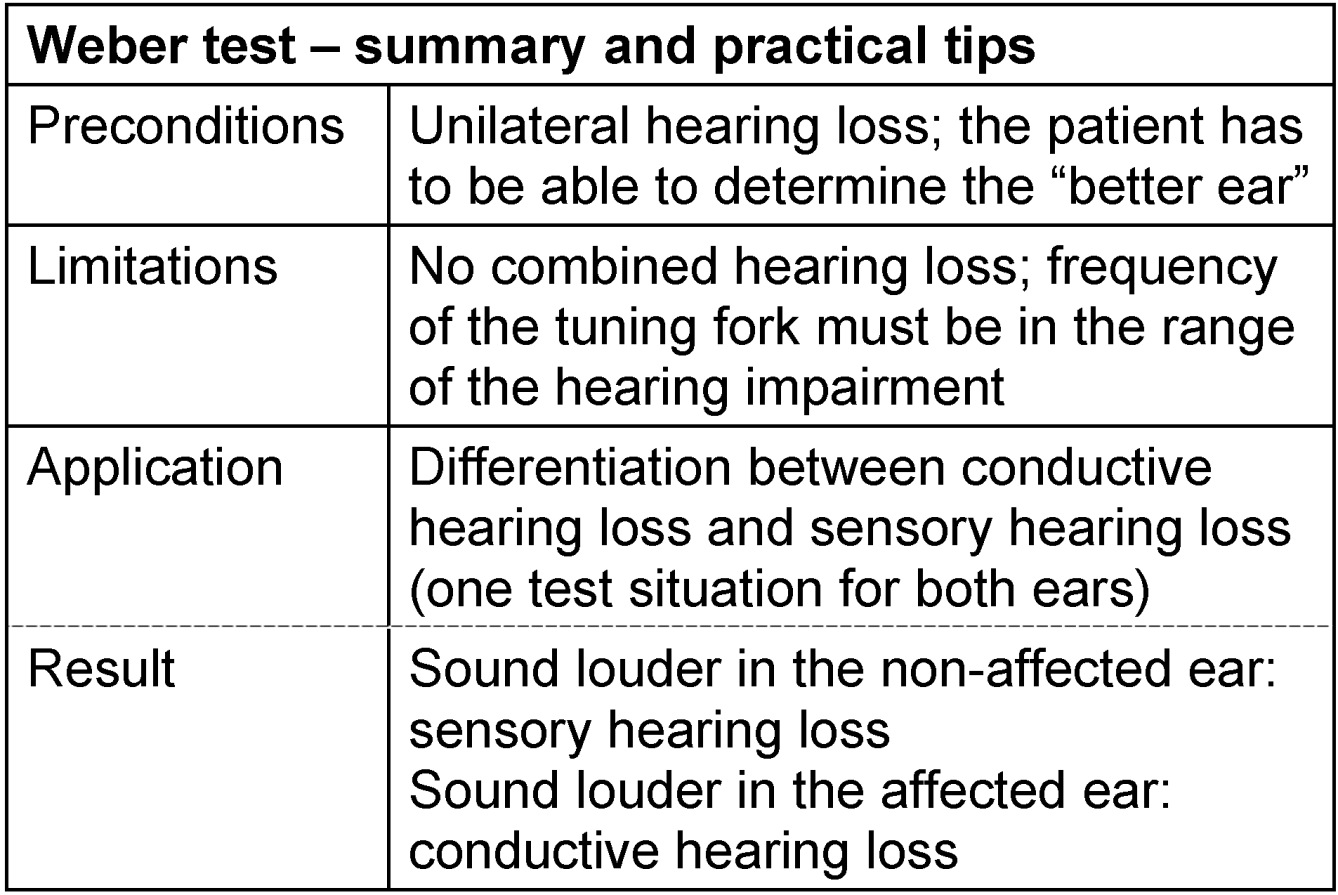
Weber test – summary and practical tips

**Table 2:**
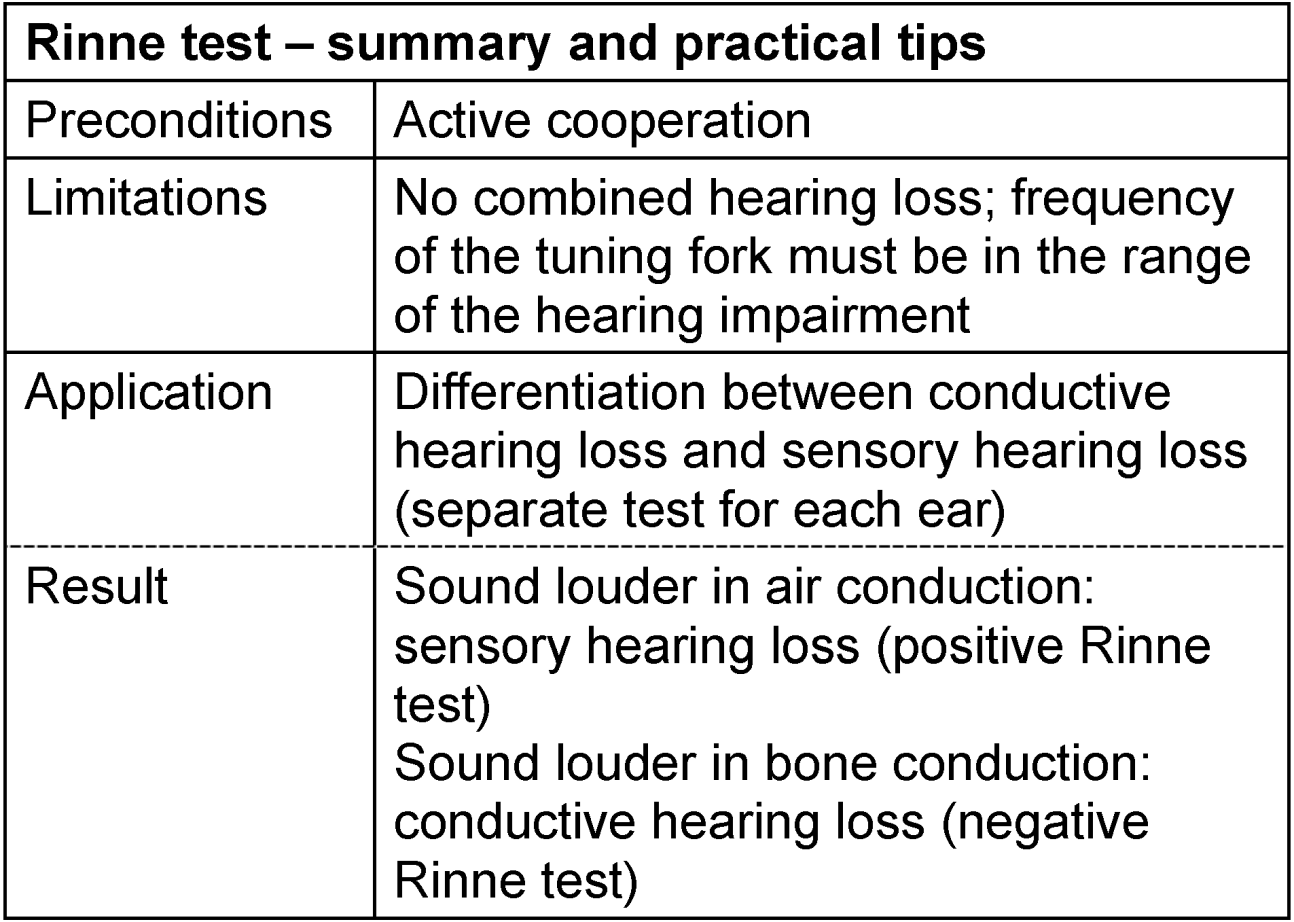
Rinne test – summary and practical tips

**Table 3:**
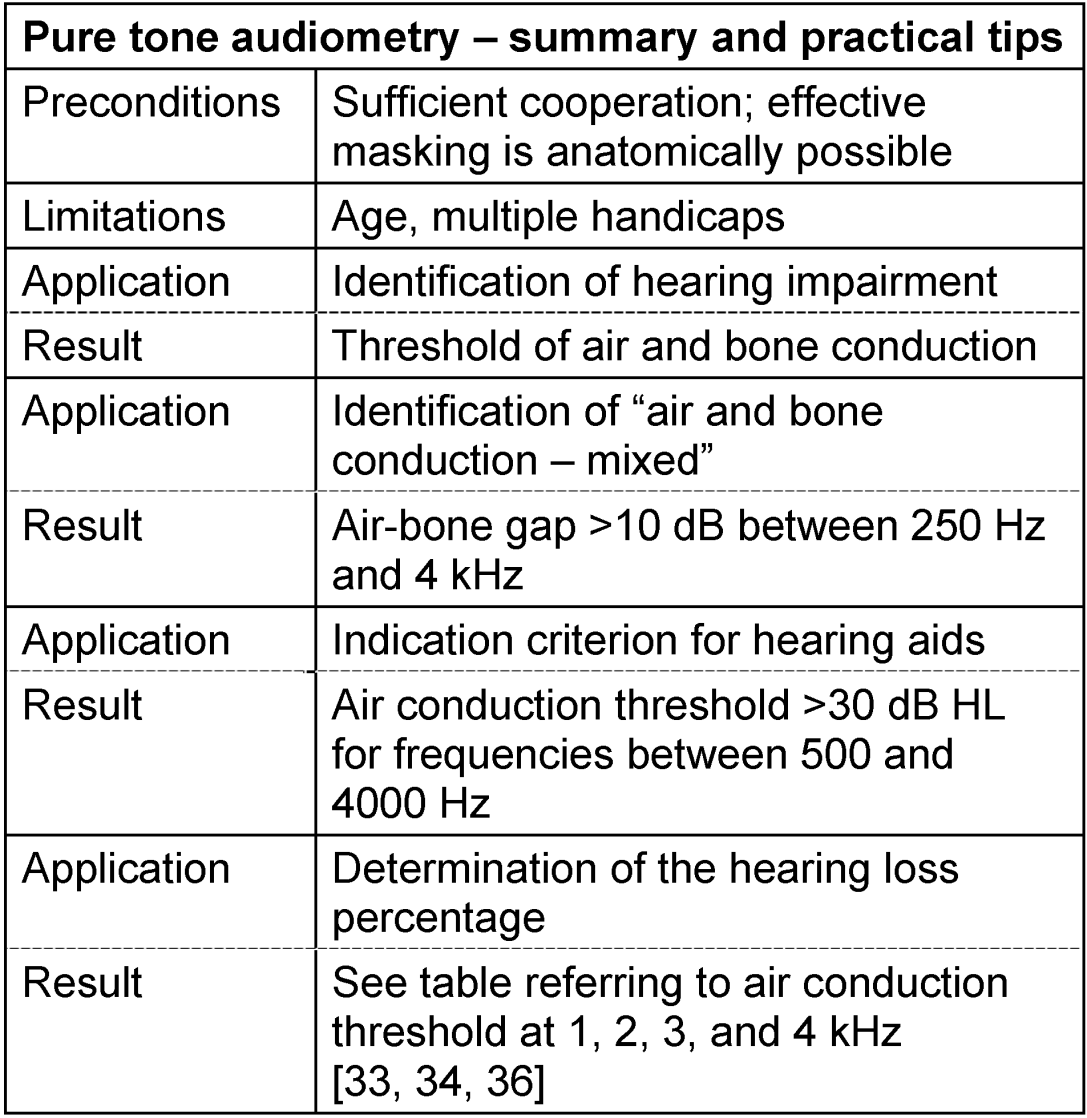
Pure tone audiometry – summary and practical tips

**Table 4:**
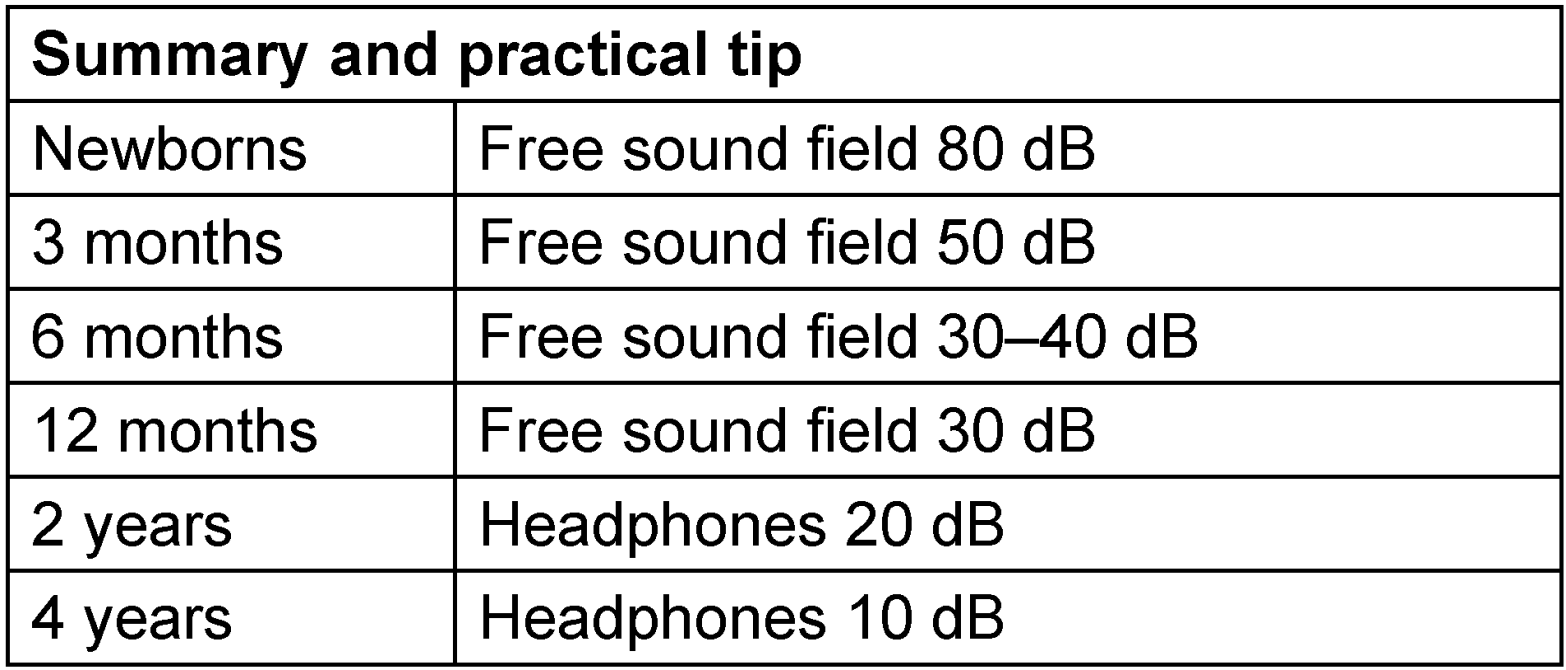
Summary and practical tip

**Table 5:**
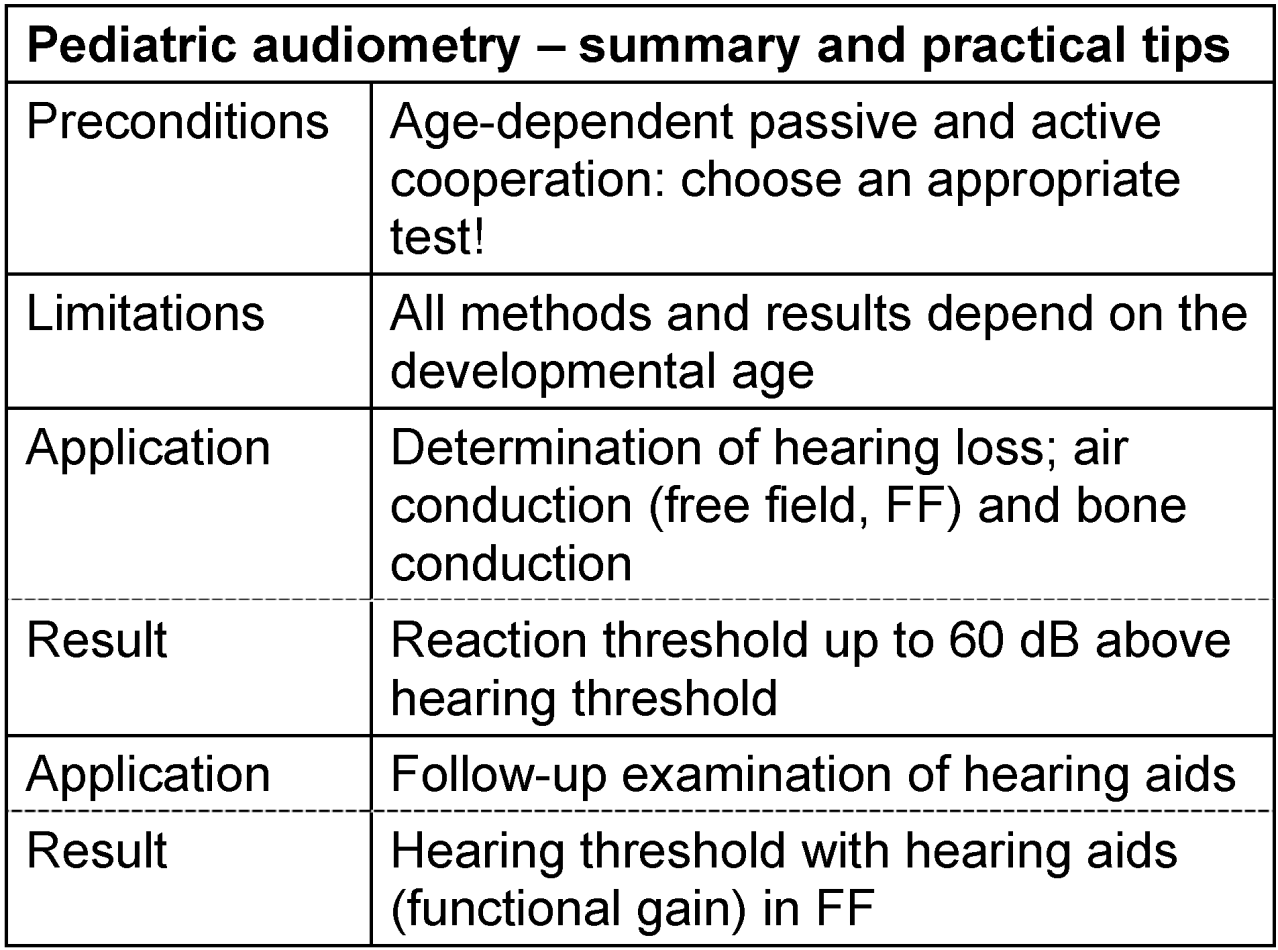
Pediatric audiometry – summary and practical tips

**Table 6:**
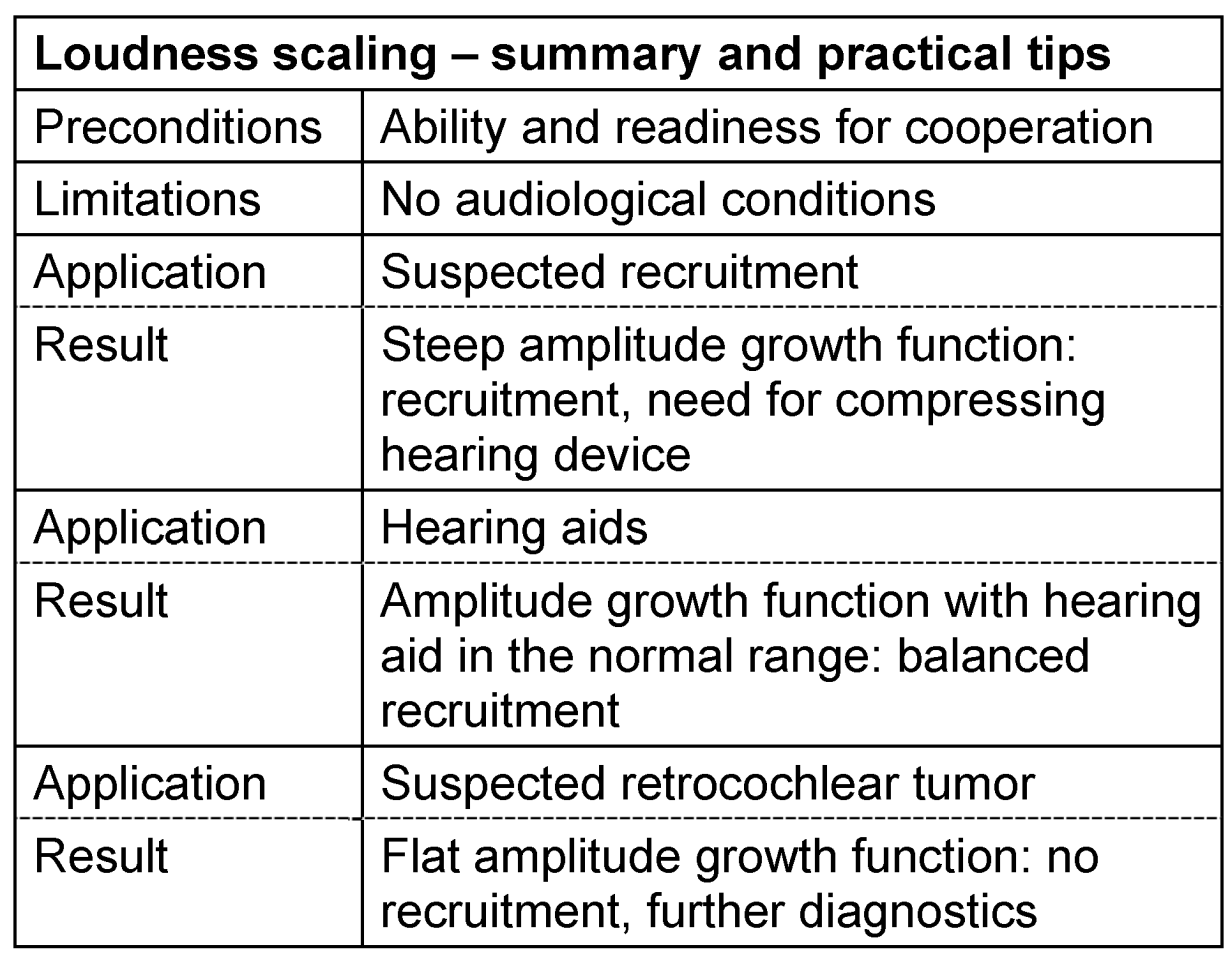
Loudness scaling – summary and practical tips

**Table 7:**
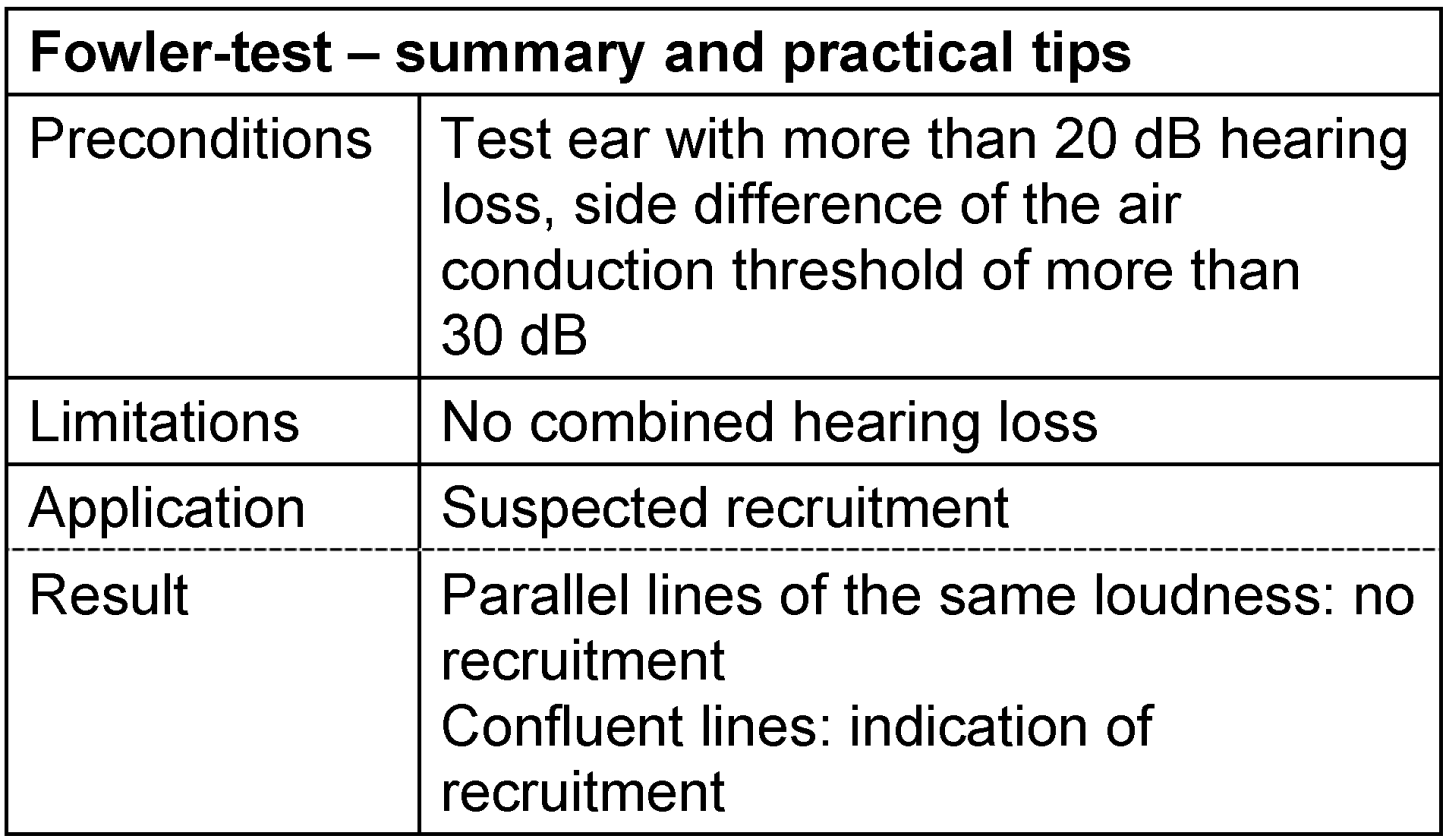
Fowler-test – summary and practical tips

**Table 8:**
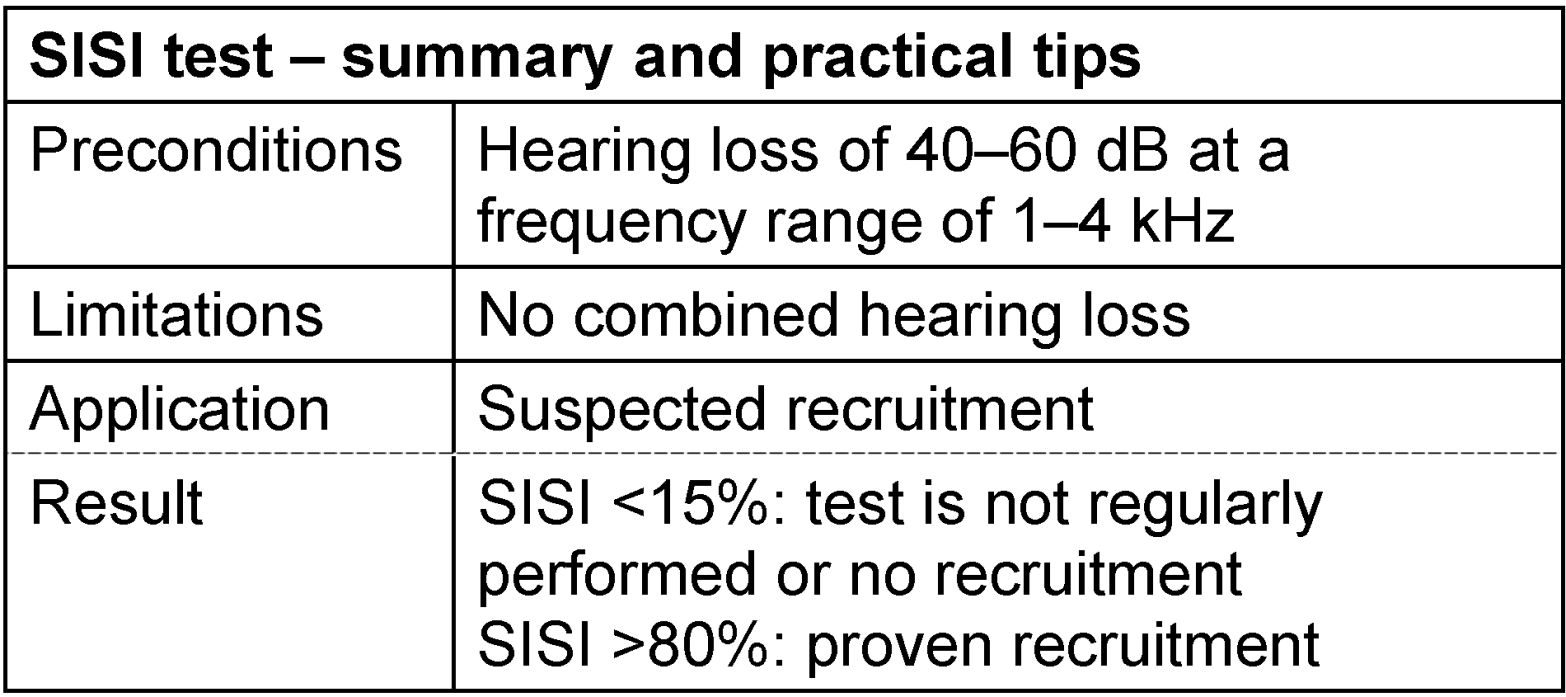
SISI test – summary and practical tips

**Table 9:**
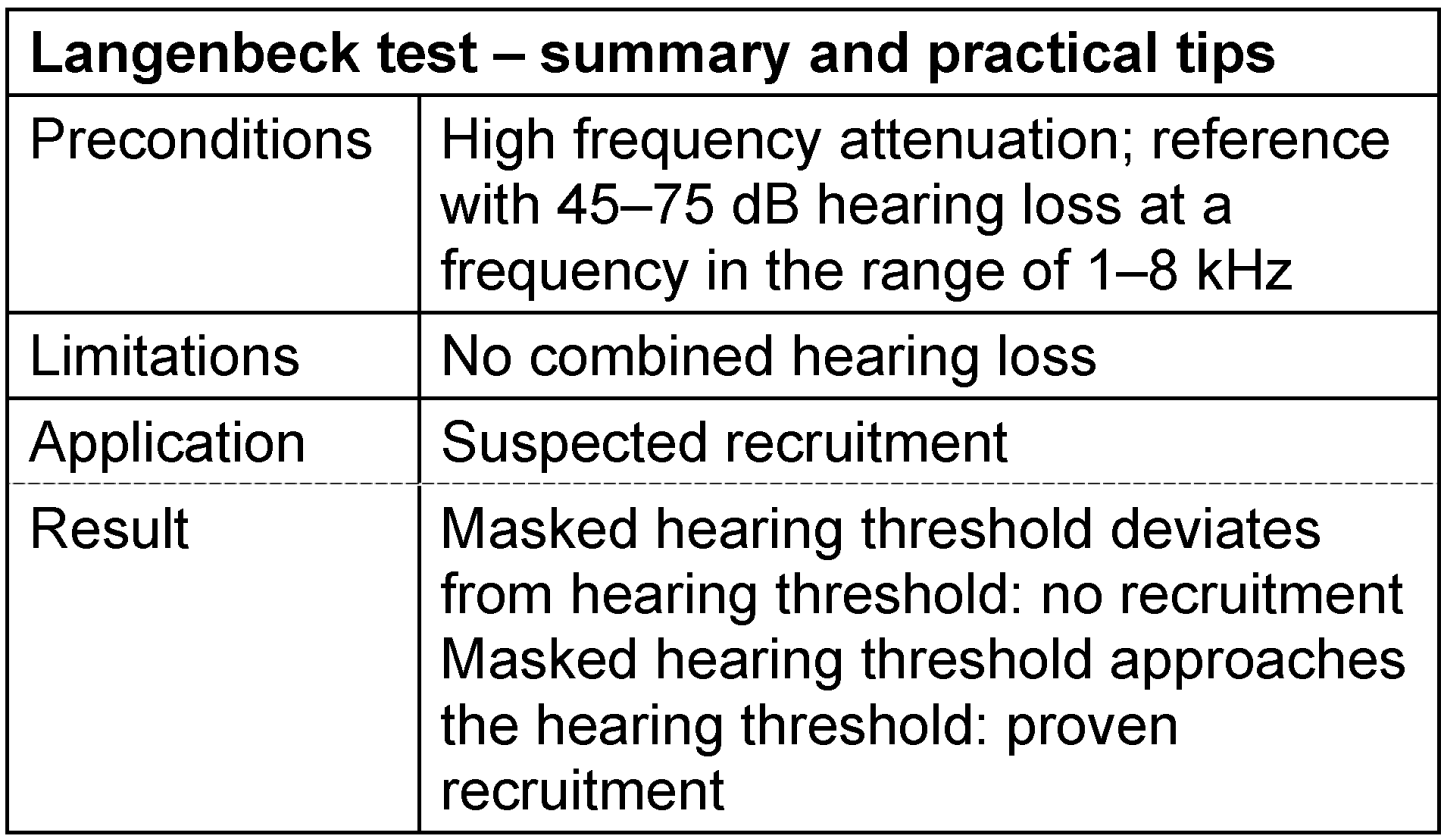
Langenbeck test – summary and practical tips

**Table 10:**
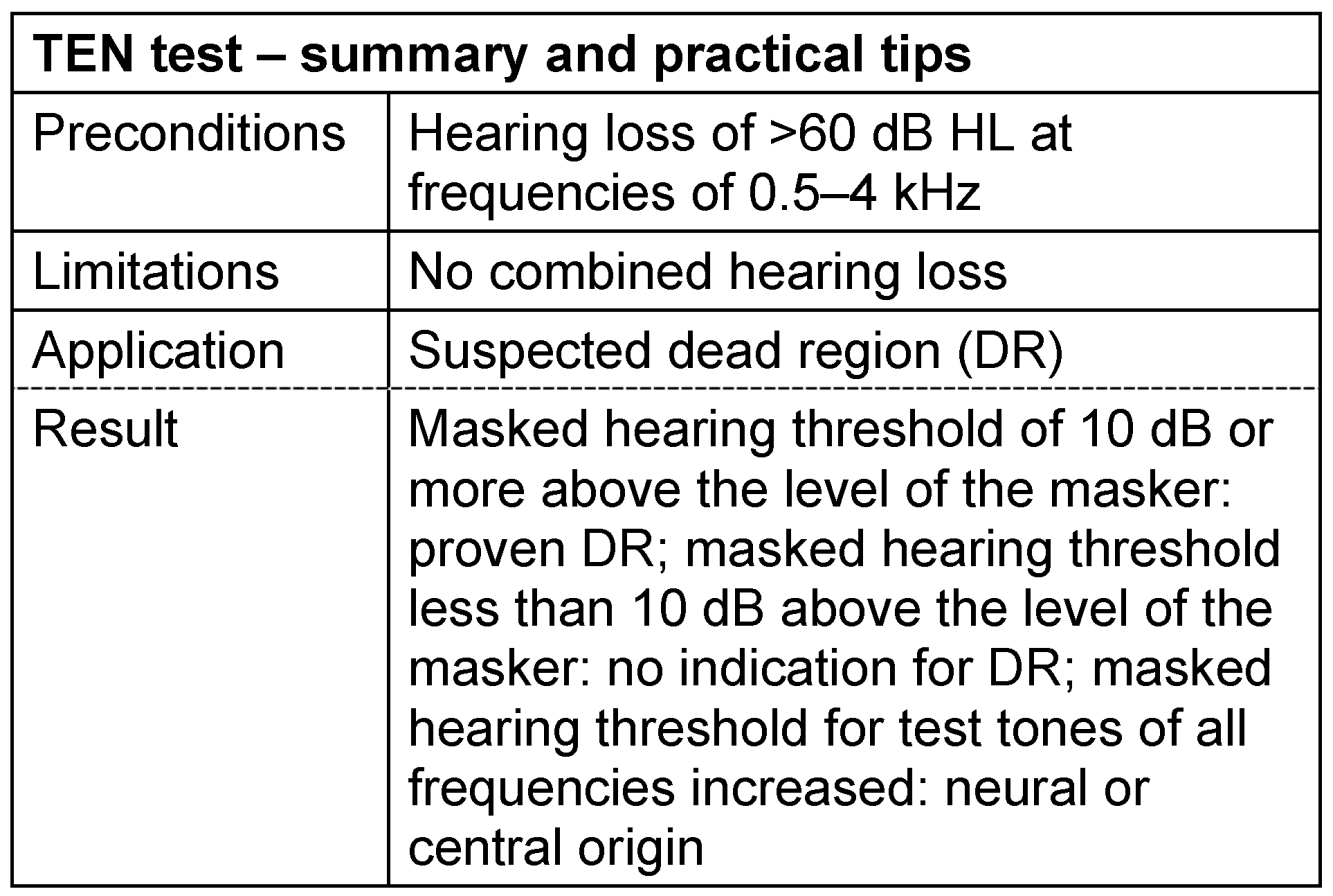
TEN test – summary and practical tips

**Table 11:**
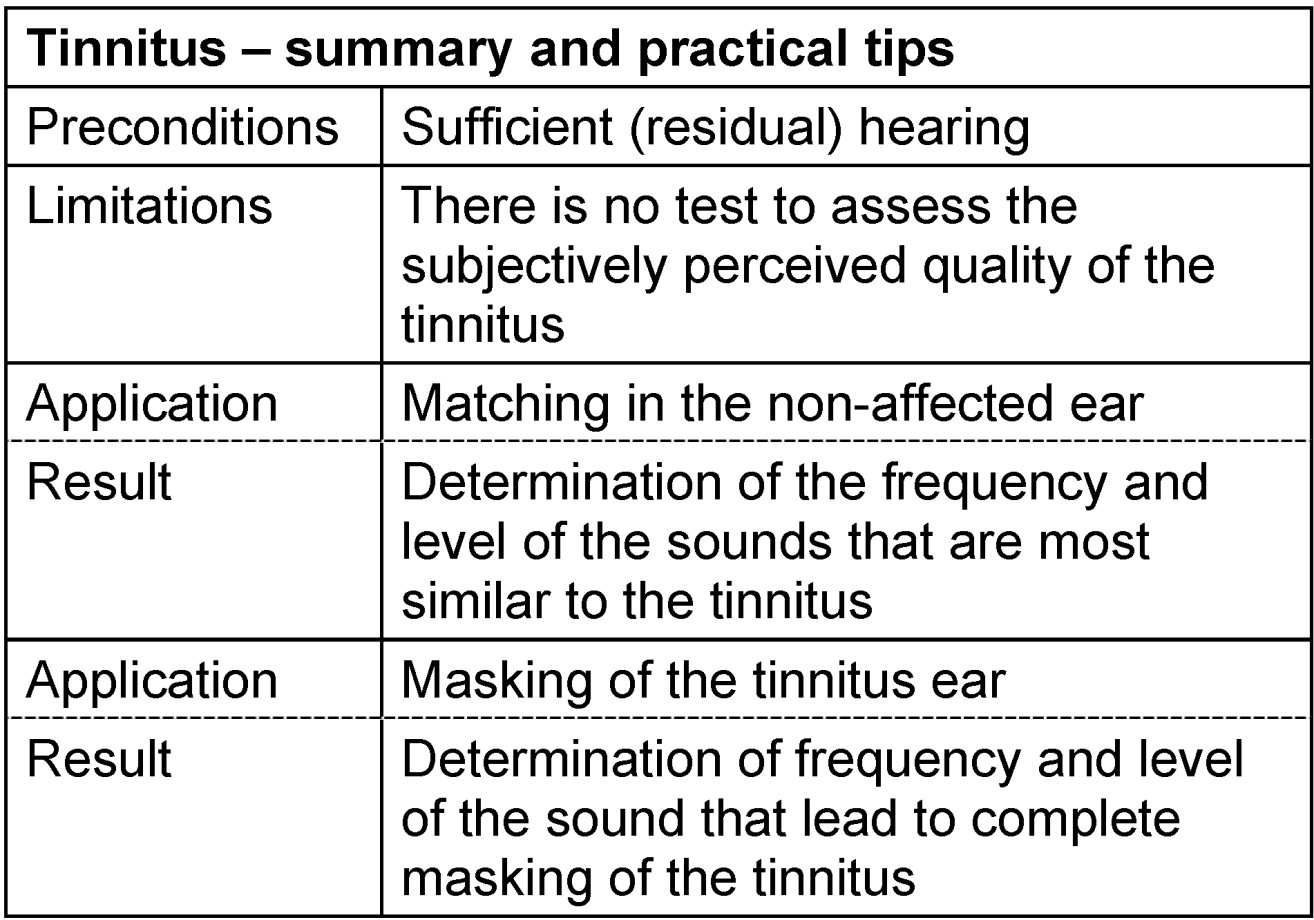
Tinnitus – summary and practical tips

**Table 12:**
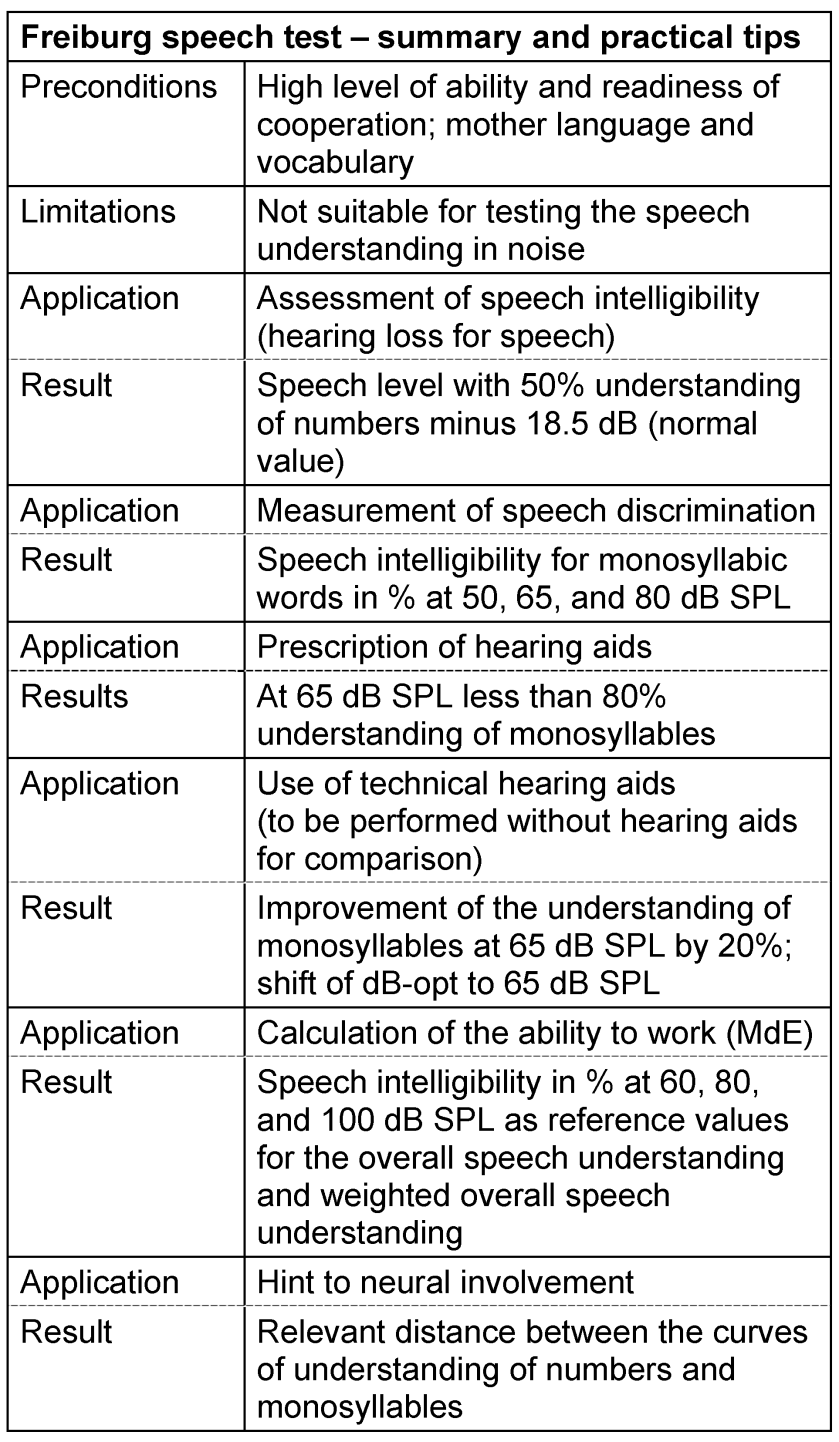
Freiburg speech test – summary and practical tips

**Table 13:**
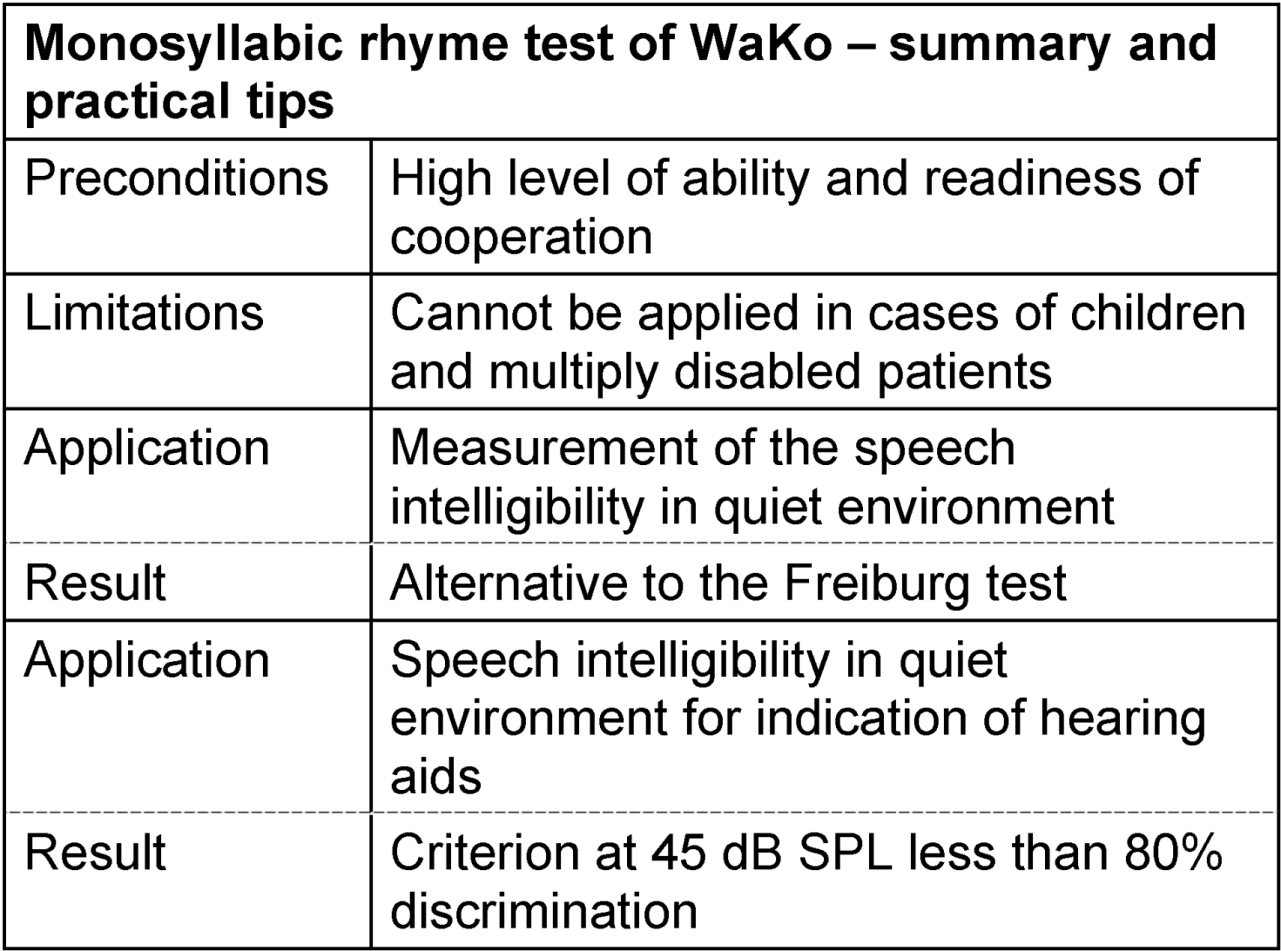
Monosyllabic rhyme test of WaKo – summary and practical tips

**Table 14:**
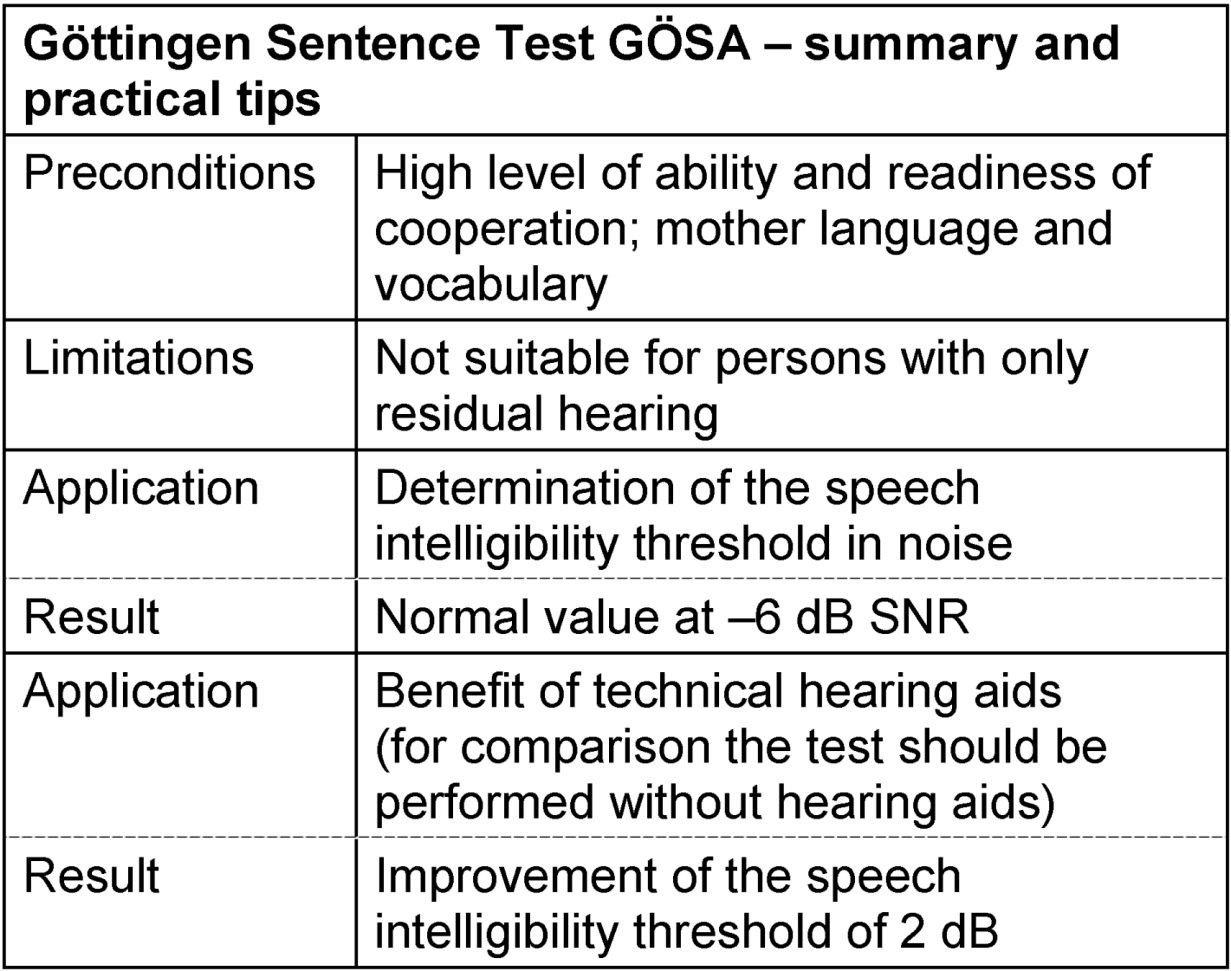
Göttingen Sentence Test GÖSA – summary and practical tips

**Table 15:**
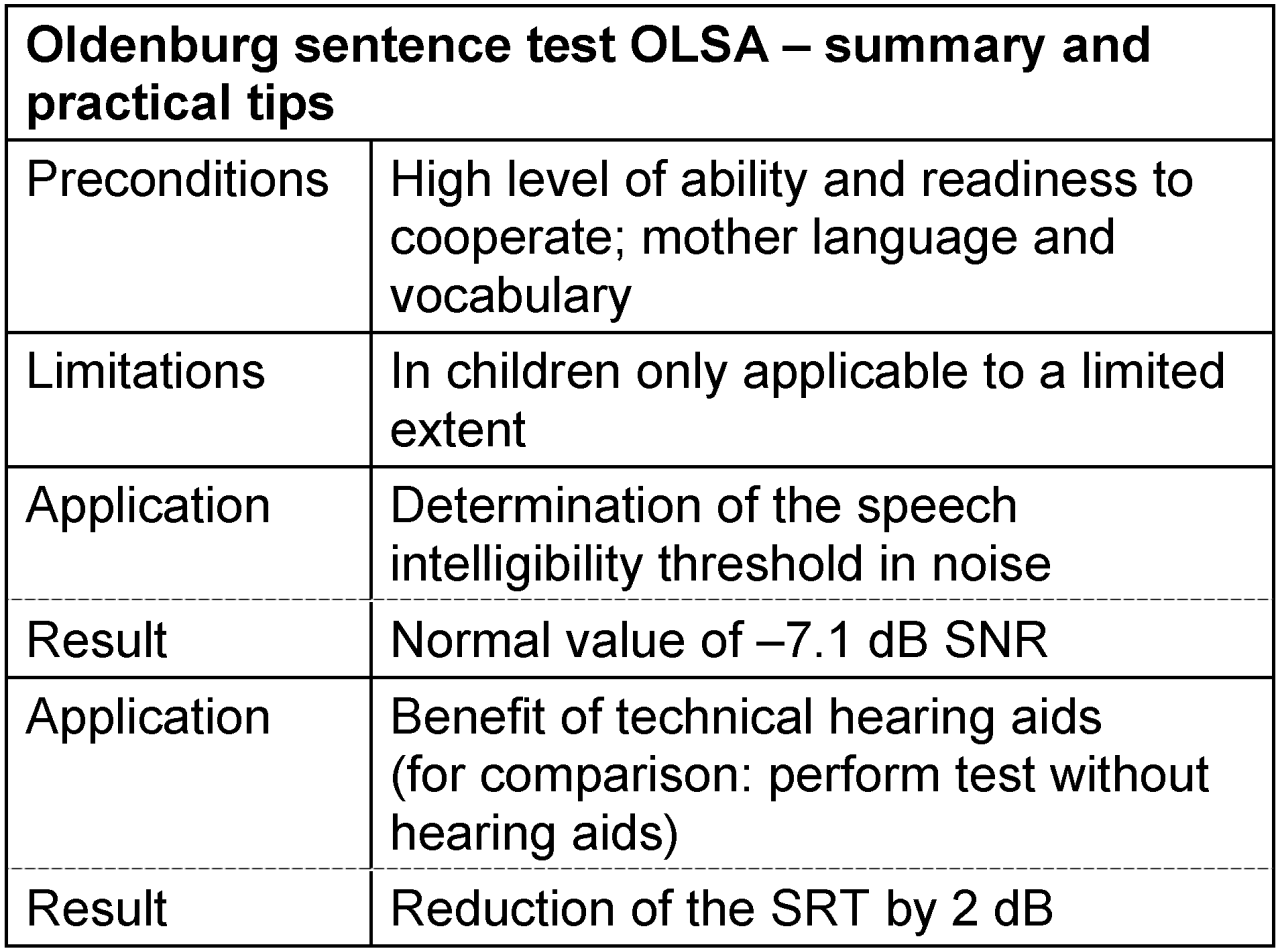
Oldenburg sentence test OLSA – summary and practical tips

**Table 16:**
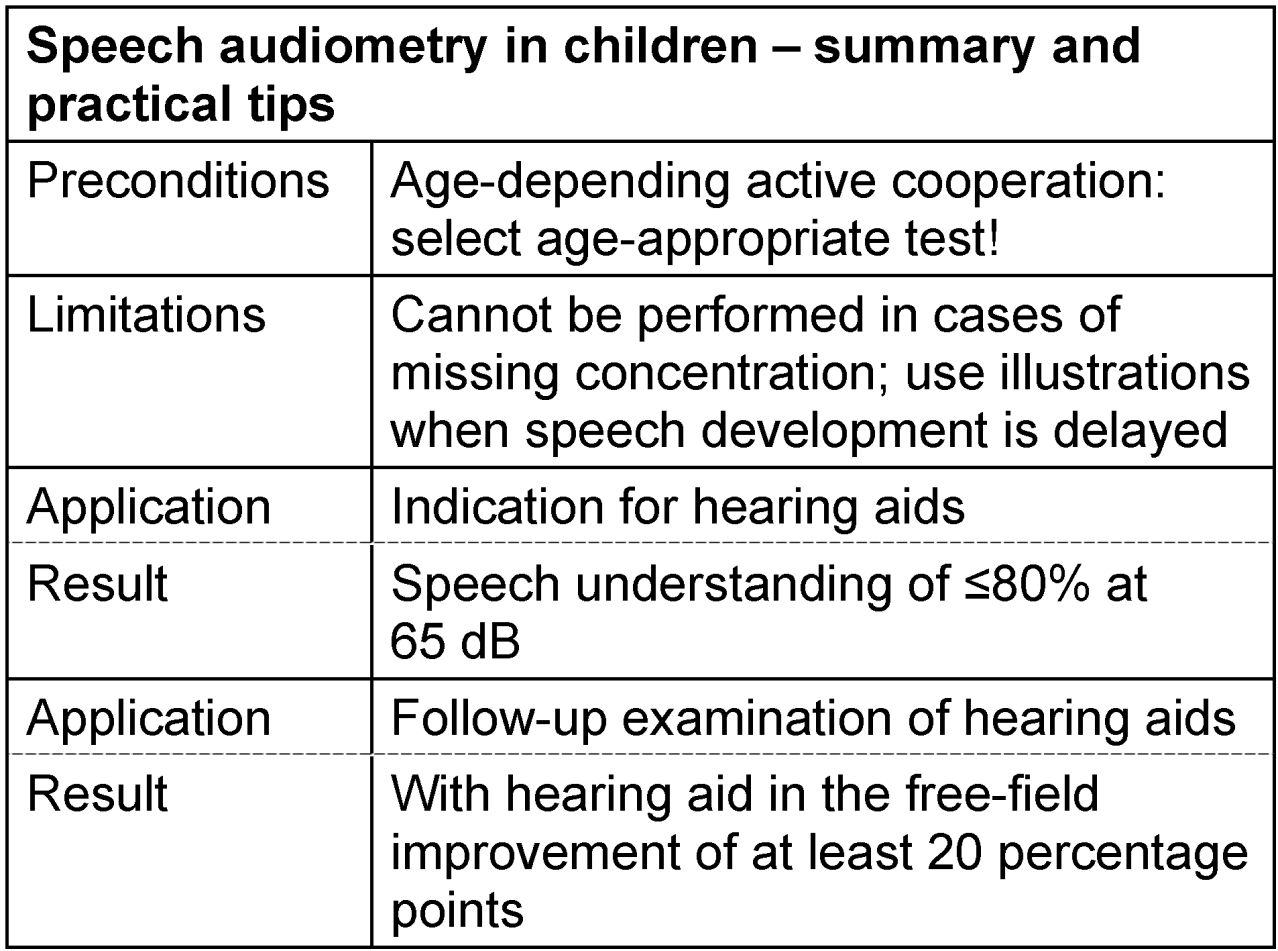
Speech audiometry in children – summary and practical tips

**Table 17:**
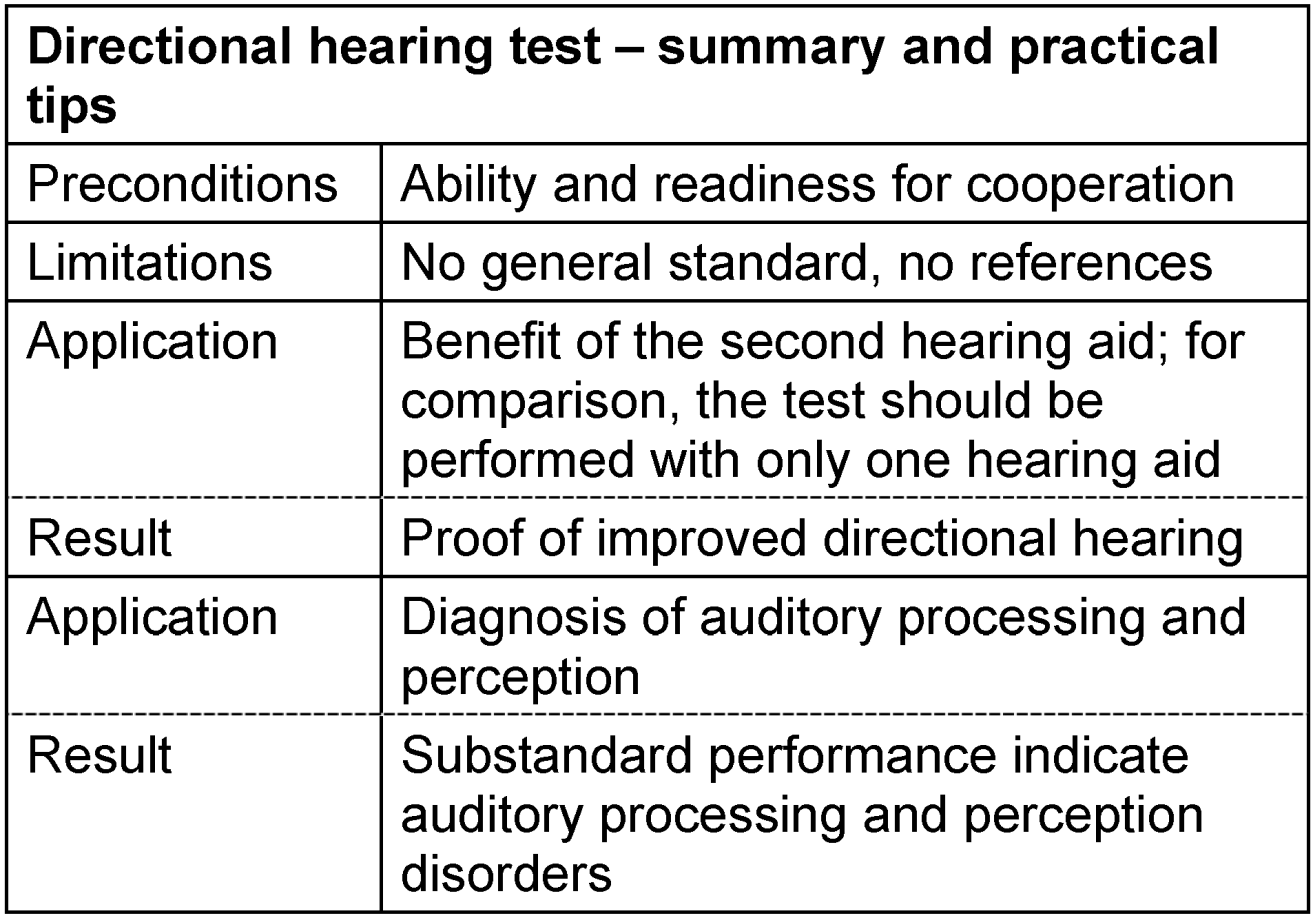
Directional hearing test – summary and practical tips

**Table 18:**
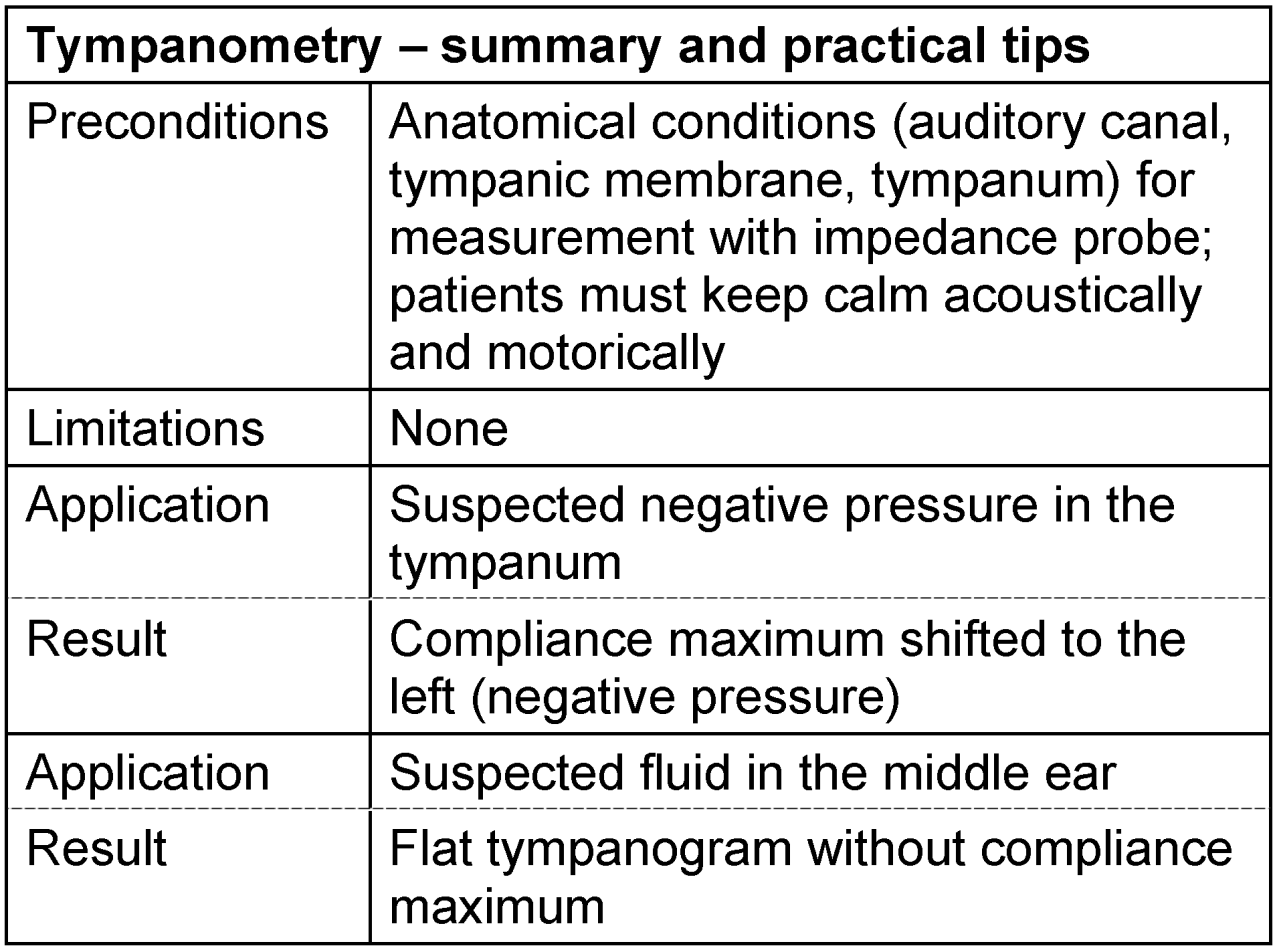
Tympanometry – summary and practical tips

**Table 19:**
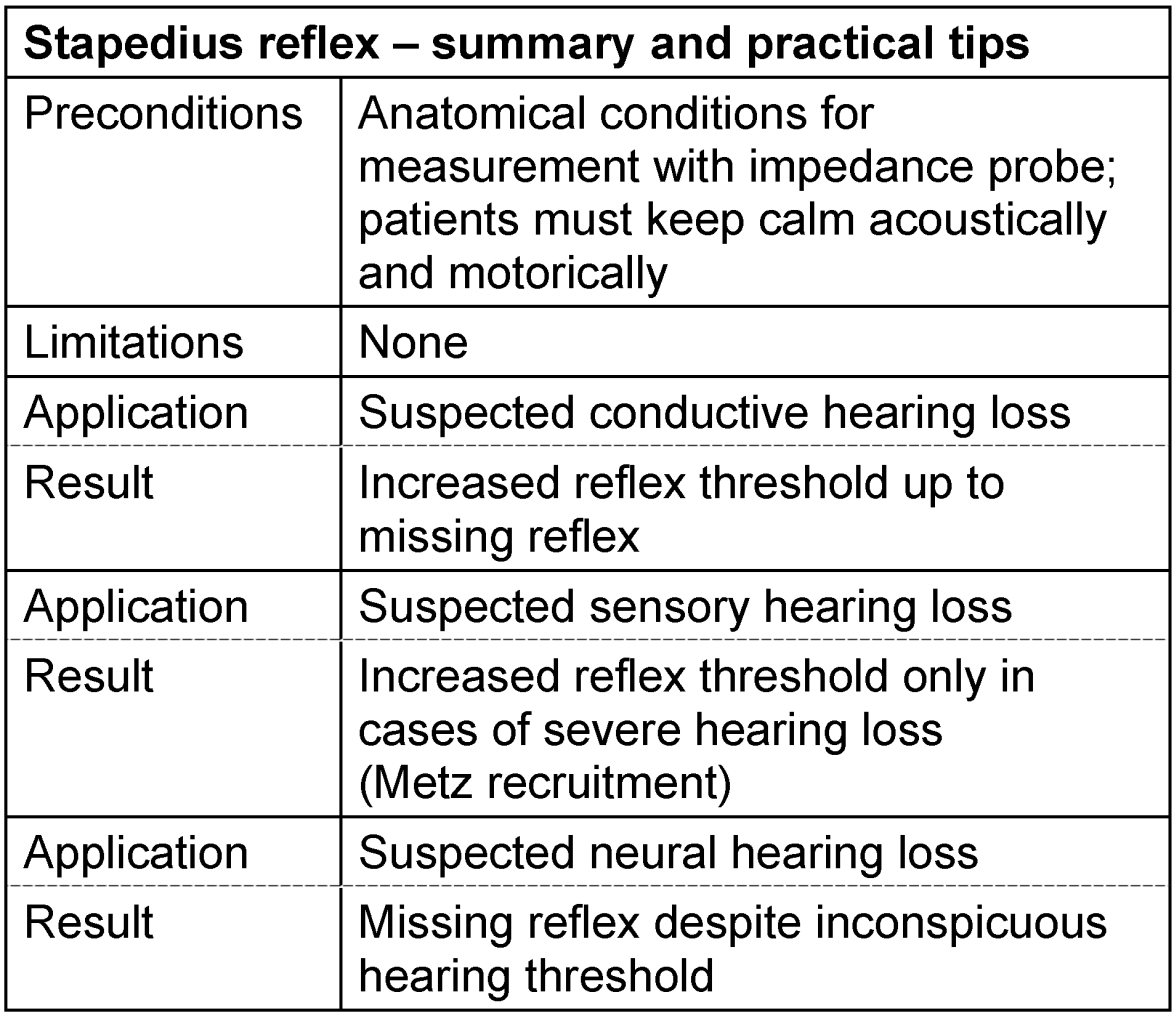
Stapedius reflex – summary and practical tips

**Table 20:**
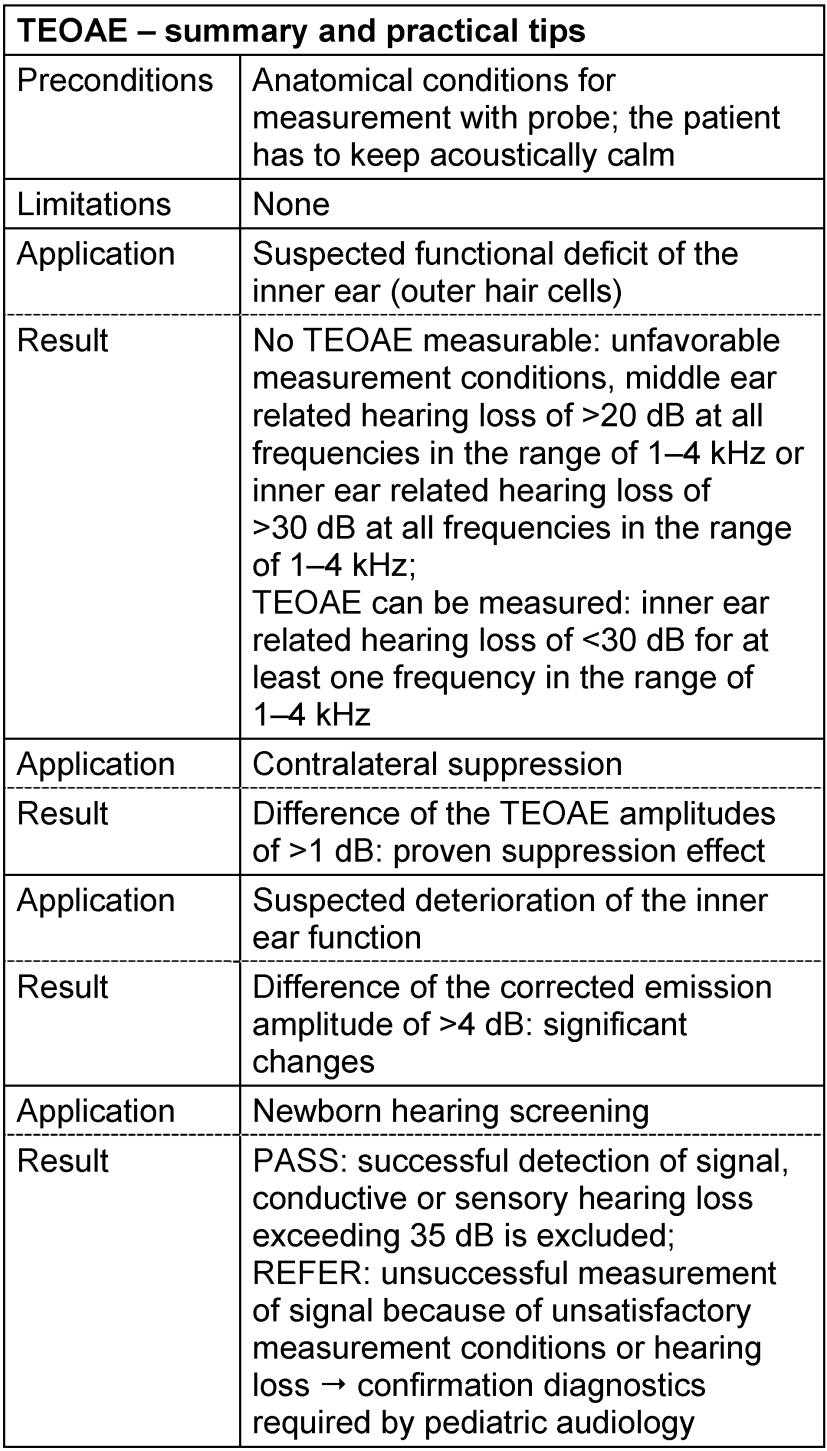
TEOAE – summary and practical tips

**Table 21:**
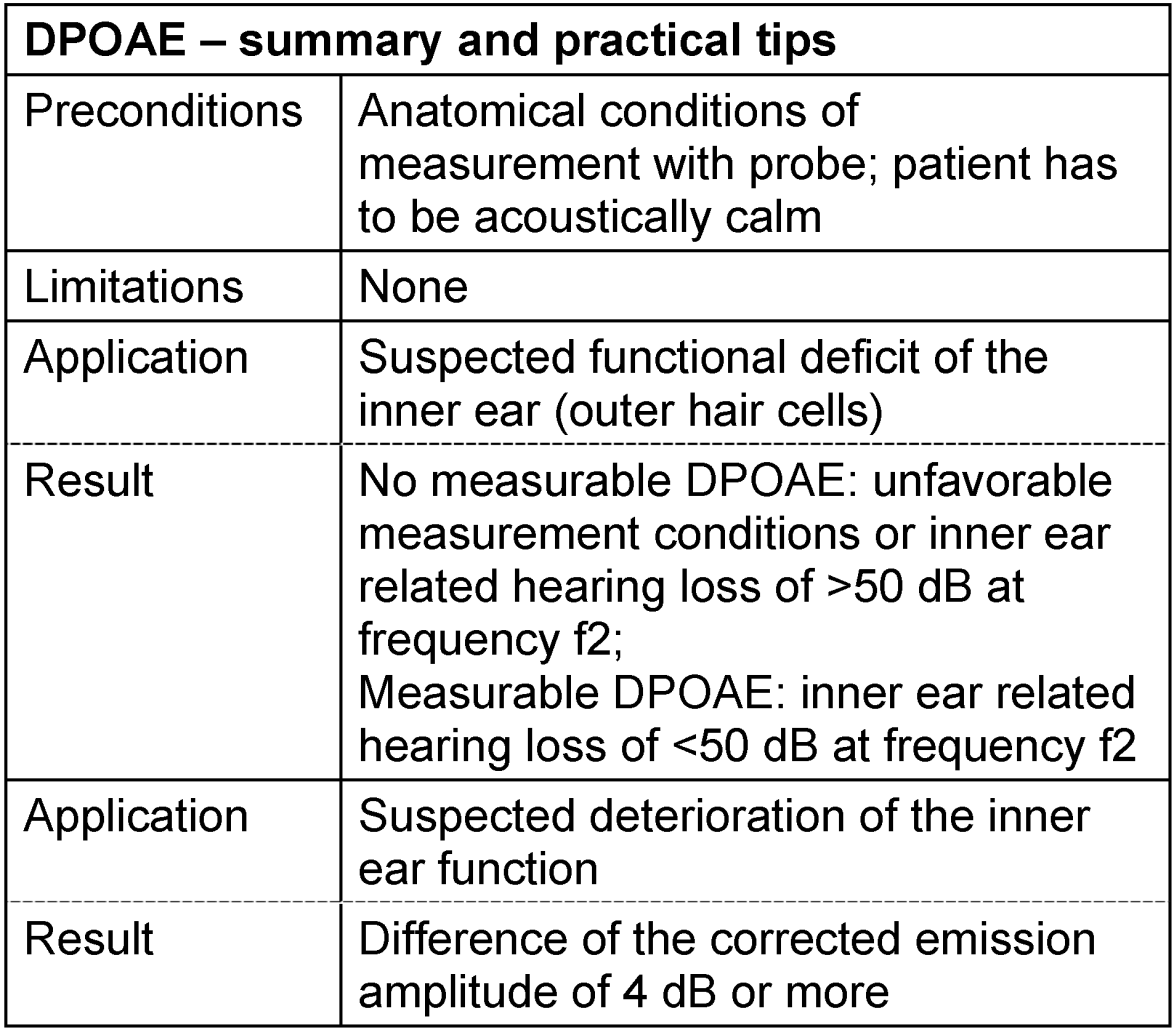
DPOAE – summary and practical tips

**Table 22:**
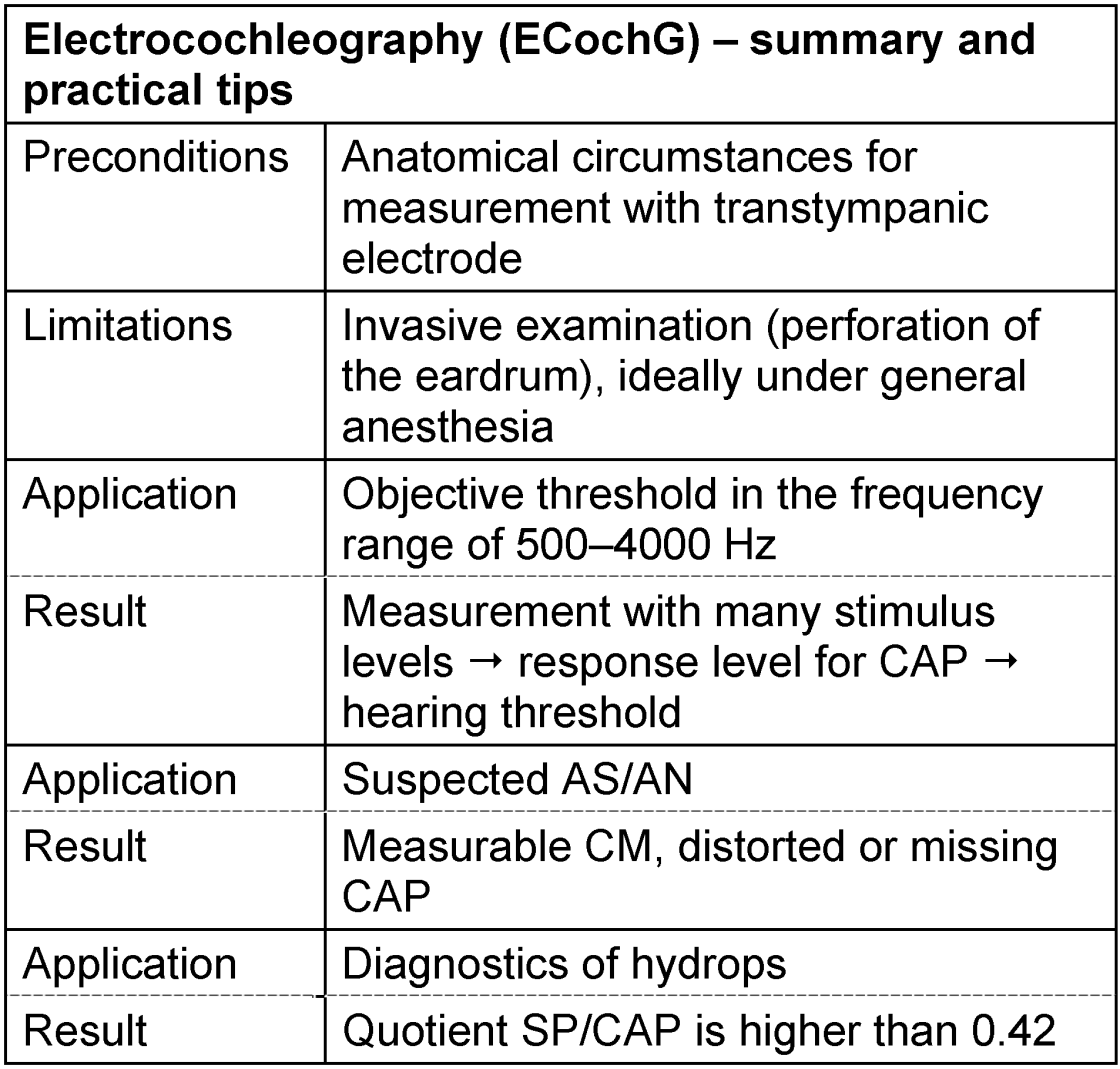
Electrocochleography (ECochG) – summary and practical tips

**Table 23:**
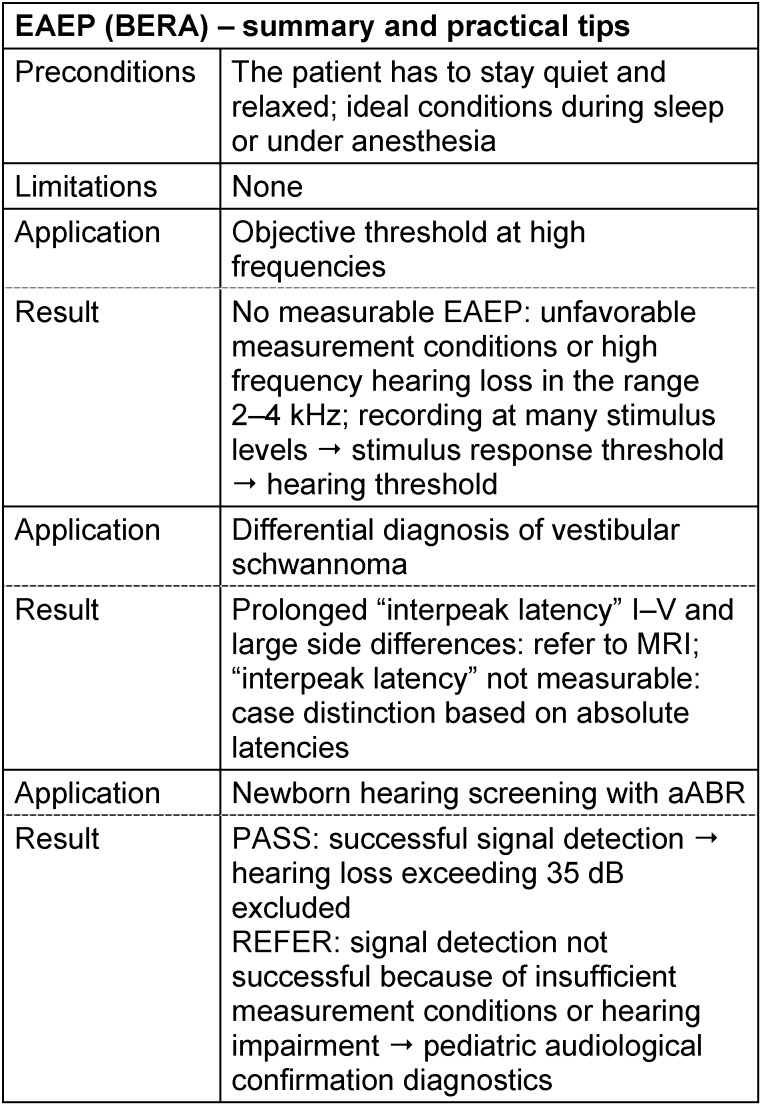
EAEP (BERA) – summary and practical tips

**Table 24:**
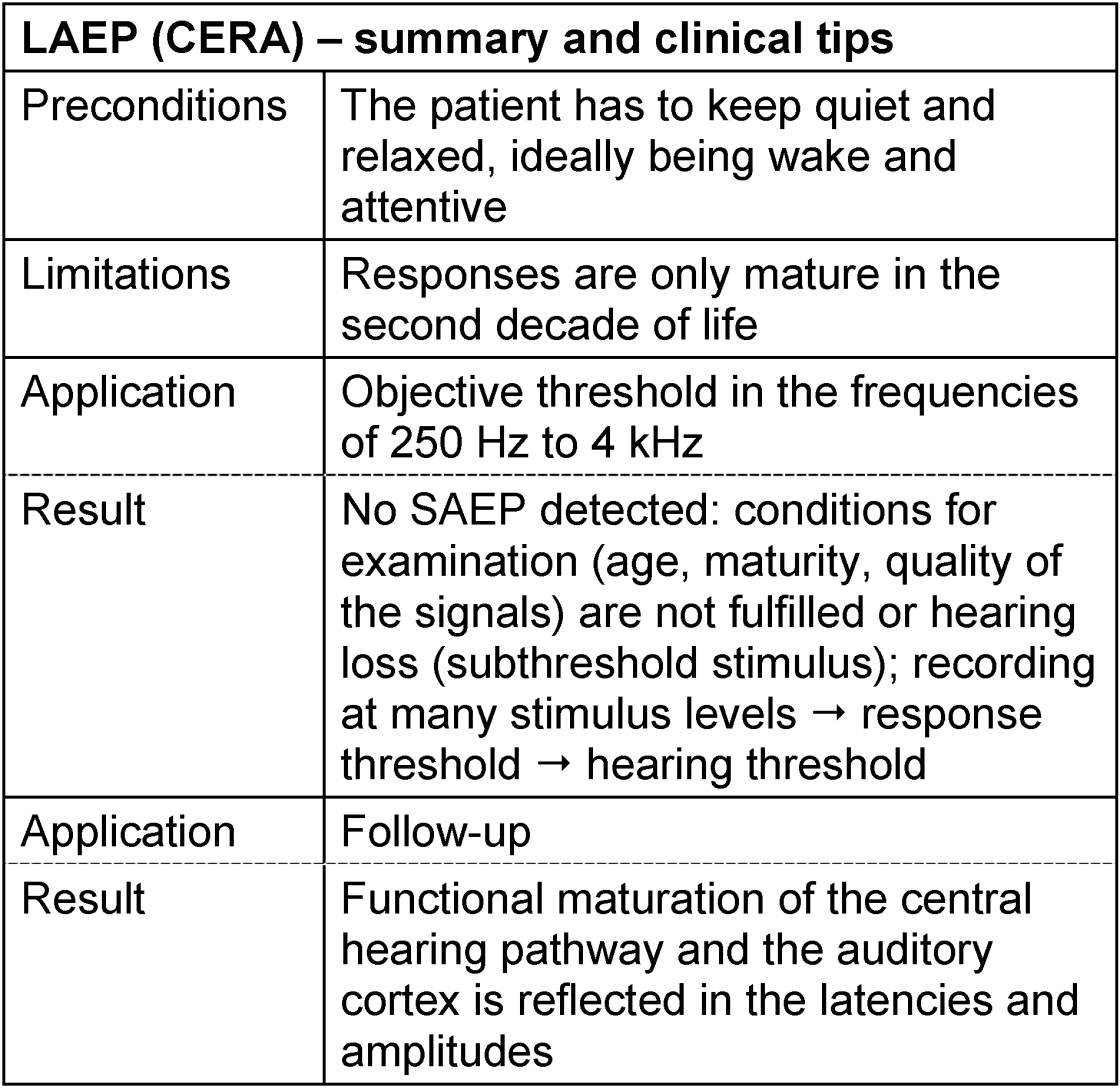
LAEP (CERA) – summary and clinical tips

**Table 25:**
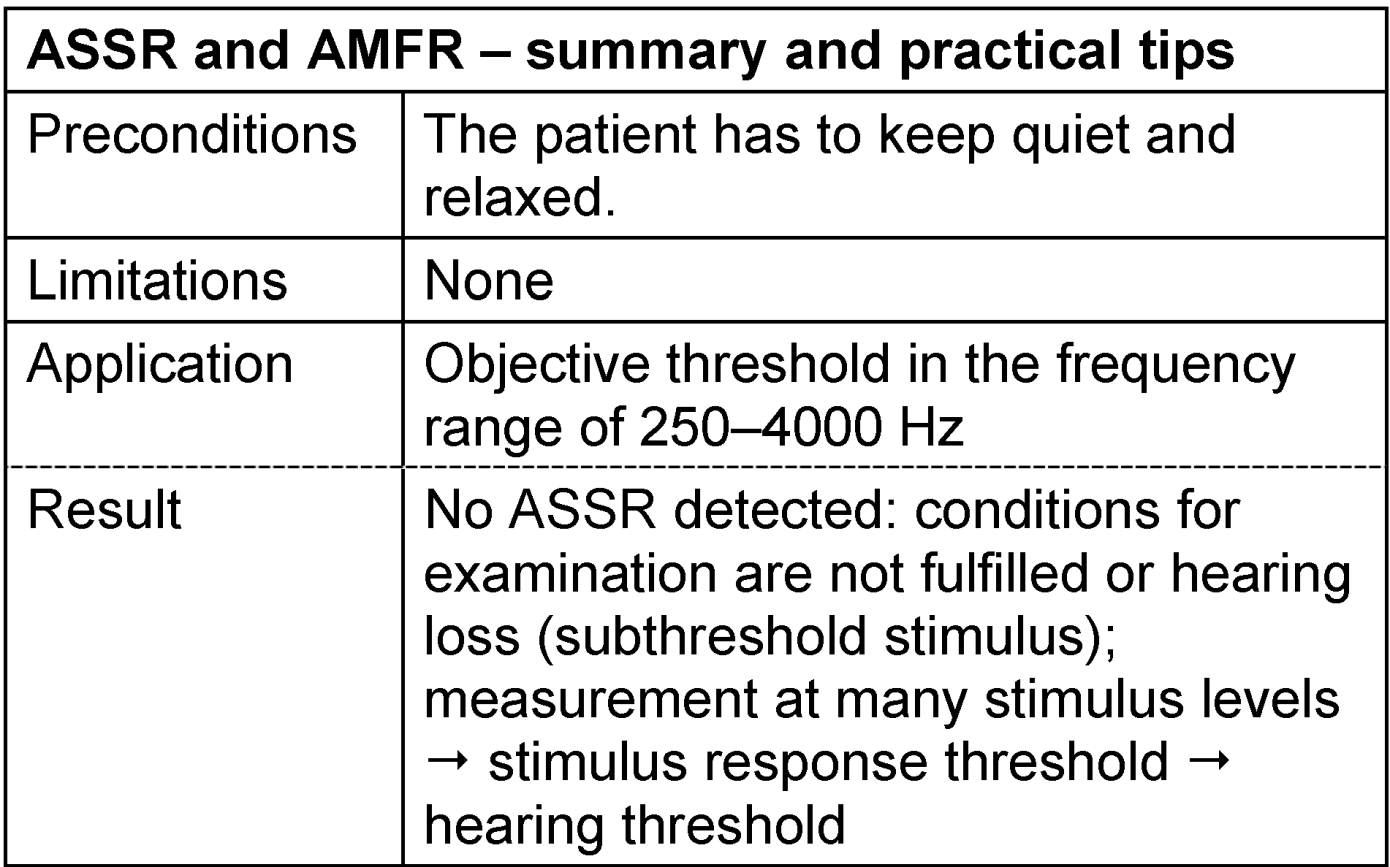
ASSR and AMFR – summary and practical tips
